# A hybrid transformer-BiLSTM model optimized with Firefly Algorithm for network traffic anomaly detection

**DOI:** 10.1371/journal.pone.0341920

**Published:** 2026-06-17

**Authors:** Debiao Luo, Weijie Wang, Xinyue Liu, Wen Yang, Ke Hu, Jia Zhang

**Affiliations:** 1 Information Network Center, Chengdu University, Chengdu, China; 2 Library, Chengdu University, Chengdu, China; Leibniz University Hannover, GERMANY

## Abstract

Network Traffic Anomaly Detection (NTAD) is essential for proactive cyber defense against increasingly sophisticated threats. This paper presents a data-driven framework that integrates adaptive signal decomposition, a hybrid attention-recurrent architecture, and metaheuristic optimization for timely anomaly prediction. Raw traffic sequences are first preprocessed via Empirical Mode Decomposition (EMD) to mitigate non-stationarity and suppress noise, yielding denoised intrinsic mode functions. The refined signal is then modeled by a hybrid deep network that couples a multi-head self-attention mechanism—capturing global, long-range dependencies—with a Bidirectional Long Short-Term Memory (BiLSTM) network that encodes bidirectional temporal dynamics. To circumvent the sensitivity of deep models to hyperparameter selection, the Firefly Algorithm (FA) is employed for automated, population-based optimization. Extensive evaluations on benchmark datasets demonstrate that the proposed EMD-FA-Transformer-BiLSTM model attains state-of-the-art performance, outperforms baseline and state-of-the-art models across all evaluated metrics, with statistically significant improvements in both regression error and classification *F*_1_-score.

## 1 Introduction

The rapid expansion of network infrastructure, propelled by the proliferation of Internet of Things (IoT) devices, cloud computing, and 5G technologies, has fundamentally reshaped the digital landscape [1–3]. While such advances enable unprecedented levels of connectivity and service provisioning, they concurrently broaden the attack surface, rendering networks increasingly susceptible to malicious activities including Distributed Denial-of-Service (DDoS) attacks [4], data exfiltration [5], and malware propagation [6]. Against this backdrop, Network Traffic Anomaly Detection (NTAD) has become a cornerstone of contemporary cybersecurity frameworks [7–10]. NTAD aims to identify deviations from established behavioral baselines, which frequently constitute early indicators of security breaches or incipient performance degradation. The capacity to furnish timely alerts for anomalous traffic fluctuations—whether abrupt surges characteristic of volumetric attacks or atypical declines signaling service disruption—is thus a strategic imperative for preserving the availability, integrity, and confidentiality of modern networked systems.

Reliable and timely anomaly prediction remains a formidable challenge, owing principally to the intrinsic characteristics of network traffic data [11]. Real-world traffic exhibits pronounced non-stationarity [12] and nonlinearity [13], with statistical moments evolving continuously under the influence of diurnal cycles, heterogeneous user behaviors, and dynamic network conditions. The signal is further corrupted by ambient noise and manifests complex, multi-scale structures [14], wherein transient bursts are superimposed upon gradual trends. Conventional detection methodologies—encompassing statistical process control [15] and signature-based Intrusion Detection Systems (IDS) [16]-are ill-equipped to contend with such intricacy. These approaches tend to be reactive in nature, exhibit limited adaptability to emerging attack vectors, and fail to adequately model the sophisticated temporal dependencies and non-stationary dynamics inherent in contemporary network traffic.

The emergence of machine learning [17–20] and, more recently, deep learning has opened promising avenues for overcoming these limitations [21–24]. Both supervised and unsupervised paradigms are capable of extracting complex patterns from historical observations. Notably, deep recurrent architectures such as Long Short-Term Memory (LSTM) and Gated Recurrent Unit (GRU) networks have demonstrated considerable efficacy in sequential modeling tasks, owing to their capacity for capturing temporal dependencies [25–29]. Nonetheless, these models are not without limitations. Their inherently sequential and localized processing renders them less adept at capturing long-range, global correlations spanning extended temporal horizons—an essential requirement for detecting stealthy, low-and-slow attack patterns. For instance, [30] introduces an RCLSTM architecture with stochastic neuron connectivity to alleviate the computational burden of conventional LSTM while preserving competitive time-series forecasting accuracy, thereby enhancing suitability for latency- or resource-constrained deployments such as telecommunications. Similarly, [31] demonstrates that LSTM networks can successfully forecast equity price movements and generate statistically significant excess returns over the 1992–2009 period, attributing profitability primarily to the exploitation of short-term reversal signals in high-volatility stocks. In [32], an LSTM-based framework is proposed for Remaining Useful Life (RUL) estimation in dynamical systems, wherein raw sensor streams are transformed into a health indicator, achieving superior degradation tracking accuracy as validated on the NASA C-MAPSS dataset. Furthermore, the performance of deep learning models is critically contingent upon hyperparameter selection, rendering manual tuning both inefficient and suboptimal in practice.

To address the challenge of non-stationarity, advanced signal processing techniques have been incorporated as a preprocessing stage [33]. Empirical Mode Decomposition (EMD) constitutes a fully data-driven methodology expressly suited for the analysis of nonlinear and non-stationary signals [34,35]. In contrast to Fourier or wavelet transforms, which rely on fixed basis functions, EMD adaptively decomposes a complex signal into a finite ensemble of Intrinsic Mode Functions (IMFs) and a monotonic residual trend. This decomposition segregates the original series into components spanning distinct temporal scales, thereby isolating high-frequency noise, transient fluctuations, and sustained long-term trends. When applied to network traffic, EMD facilitates denoising, uncovers inherent multi-scale structures, and transforms a non-stationary sequence into a collection of comparatively stationary sub-components, yielding a more tractable and informative representation for downstream predictive modeling. For instance, [36] proposes a hybrid MEMD-TCN framework for stock index forecasting, wherein Multivariate EMD is employed to process multi-indicator financial data, and Temporal Convolutional Networks perform the prediction task, achieving enhanced accuracy and robustness across diverse national market indices. In [37], a Fast and Adaptive EMD (FAEMD) variant is introduced, integrating an Order Statistics Filter to efficiently construct signal envelopes and extract fault-related features, thereby attaining reduced computational overhead and competitive diagnostic performance relative to conventional EMD implementations.

On the architectural front, the Transformer model [38], built on a multi-head self-attention mechanism [39], has revolutionized sequence modeling. It excels at modeling global contextual relationships by allowing every element in a sequence to interact directly with all others, irrespective of distance. This makes it exceptionally capable of identifying correlations between widely spaced events in a traffic stream. When combined with a Bidirectional Long Short-Term Memory (BiLSTM) network—a model renowned for its proficiency in learning ordered, bidirectional temporal dependencies—a powerful hybrid architecture is formed [40–42]. This hybrid model can leverage the Transformer’s strength in capturing global patterns and the BiLSTM’s finesse in modeling localized, sequential evolution. Nevertheless, building such a hybrid model introduces a complex hyperparameter optimization problem. The convergence, efficiency, and final performance of the model depend heavily on the optimal selection of these parameters. Here, nature-inspired metaheuristic algorithms offer a robust solution [43]. In [44], a hybrid LSTM-genetic algorithm (GA) model is proposed for stock market prediction, where the GA is utilized to systematically optimize the time window size and network topology of LSTM, demonstrating superior performance on the Korea Stock Price Index (KOSPI) index compared to benchmark models. In [45], a particle swarm optimization (PSO)-optimized LSTM network is proposed to address the nonlinear and noisy challenges in ship motion prediction, with testing results showing that this hybrid method effectively avoids local optima and improves prediction accuracy. The Firefly Algorithm (FA) [46], inspired by the flashing behavior and attraction of fireflies, is particularly effective for global optimization in complex search spaces [47]. It efficiently navigates the parameter landscape to find configurations that maximize model performance, automating a process that would otherwise be prohibitively time-consuming.

This paper, therefore, addresses the identified research gaps by proposing an integrated data-driven framework for network traffic anomaly prediction. The core contribution lies in the purposeful synthesis of adaptive signal preprocessing, a hybrid attention-recurrent architecture, and metaheuristic hyperparameter optimization. Specifically, our work advances the state of the art through the following three aspects:

1). We use empirical mode decomposition to adaptively decompose the original and non-stationary business sequence into inherent mode functions. This preprocessing step separates the multi-scale model from the high-frequency noise, and produces a structural representation of denoising, which significantly enhances the learnability of the downstream model.
2). A hybrid deep learning architecture is designed, which couples the transformer encoder (using multi-head self-attention to capture the global and long-distance traffic correlation) with the bidirectional LSTM network with fine-grained bidirectional time dependence. This dual-stream design supports comprehensive sequence modeling, which can not be realized by any component alone.
3). The Firefly Algorithm is employed to automate the joint optimization of critical hyperparameters within the hybrid model. As a population-based metaheuristic, FA efficiently explores the combinatorial search space, yielding configurations that reduce validation loss relative to manually tuned baselines.

The remainder of this paper is organized as follows: Section 2 reviews related work in network anomaly detection, signal decomposition, and hybrid deep learning models. Section 3 details the proposed methodology, including the EMD preprocessing, the architecture of the hybrid model, and the FA optimization process. Section 4 describes the experimental setup, datasets, and presents a comprehensive analysis of the results. Finally, Section 5 concludes the paper and suggests directions for future research.

## 2 Data processing and problem formulation for network traffic anomaly detection

### 2.1 Definition of anomaly detection metrics

**Fig 1:**
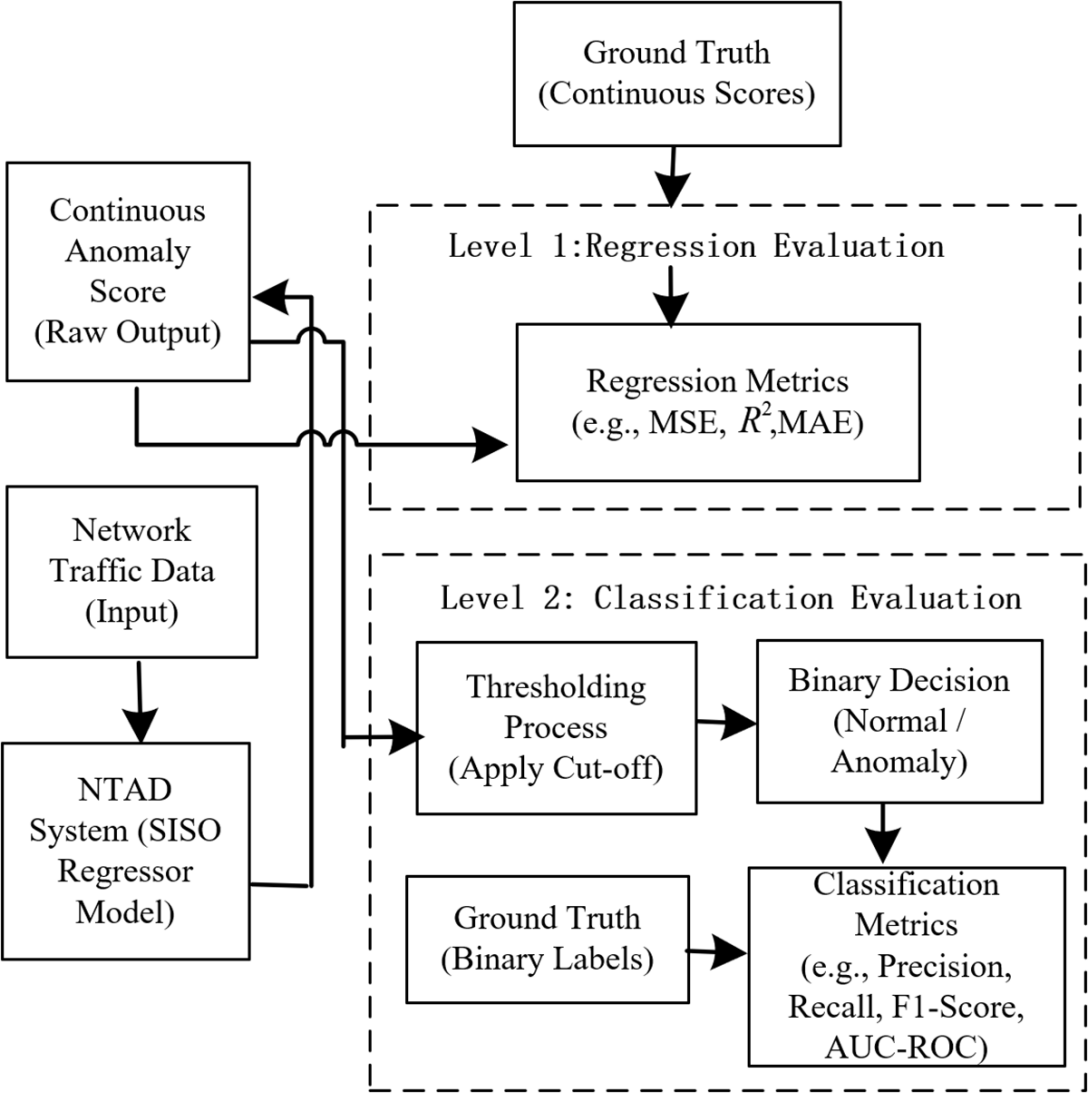
Schematic diagram of the dual-level evaluation mechanism for the SISO regressor-based NTAD system.

The performance of a NTAD system is evaluated using a combination of metrics that assess its capability to correctly identify threats while minimizing false alarms. Given that our proposed model functions as a Single-Input-Single-Output (SISO) regressor predicting a continuous anomaly score, evaluation occurs at two levels: 1) Regression performance on the raw score, and 2) Classification performance after applying a threshold to convert the score into a binary decision, as shown in Fig 1.

Let $y_{\text{true }}\in \mathbb{R}$ be the ground-truth anomaly score (where 0 typically represents “normal” and higher values indicate severity or different anomaly types) and ${\hat{y}}_{\text{pred }}\in \mathbb{R}$ be the model’s predicted score. For binary classification, a threshold τ is applied: ${\hat{y}}_{\text{binary }}=𝕀\left({\hat{y}}_{\text{pred }}\geq \tau \right)$, where 𝕀(·) is the indicator function. Based on the binary predictions, we define the following confusion matrix quantities: True Positives (TP), False Positives (FP), True Negatives (TN), and False Negatives (FN).

### 2.2 Model performance evaluation metrics

To comprehensively evaluate the proposed model, we adopt a hybrid assessment framework encompassing both regression accuracy and classification efficacy. Since the model outputs a continuous anomaly score that is subsequently thresholded to yield a binary decision (normal vs. anomalous), this dual-perspective evaluation is indispensable: regression metrics quantify the precision of the predicted score, while classification metrics assess the quality of the resulting alert. Let $y_{i}\in \mathbb{R}$ denote the ground-truth target for the *i*-th sample—interpreted as a continuous anomaly score for regression and as a binary label 0,1 for classification—and let ${\hat{y}}_{i}\in \mathbb{R}$ denote the corresponding model prediction. Given *N* evaluation samples and a predefined threshold τ, the binary prediction is obtained as ${\hat{y}}_{i}^{\text{binary}}=𝕀({\hat{y}}_{i}\geq \tau )$, where 𝕀(·) is the indicator function.

#### 2.2.1 Regression performance metrics.

These metrics quantify the accuracy of the continuous anomaly score prediction [48].

a). Mean Absolute Error (MAE)

The MAE measures the average magnitude of absolute errors between the predicted and true scores, providing a linear and interpretable measure of deviation.


$$\begin{array}{c} MAE=\frac{1}{N}\sum_{i=1}^{N}\left|y_{i}-{\hat{y}}_{i}\right| \end{array}$$ (1)

where *N* is the total number of samples in the evaluation dataset. $y_{i}$ is the ground-truth (actual) continuous anomaly score for the *i*-th sample. ${\hat{y}}_{i}$ is the predicted continuous anomaly score for the *i*-th sample generated by the model. |·| denotes the absolute value operator.

b). Root Mean Square Error (RMSE)

$$\begin{array}{c} RMSE=\sqrt{\frac{1}{N}\sum_{i=1}^{N}{\left(y_{i}-{\hat{y}}_{i}\right)}^{2}} \end{array}$$ (2)

c). Coefficient of Determination $\left(R^{2}\right)$

$$\begin{array}{c} R^{2}=1-\frac{SS_{res}}{SS_{tot}} \end{array}$$ (3)

$$\begin{array}{c} SS_{res}=\sum_{i=1}^{N}{\left(y_{i}-{\hat{y}}_{i}\right)}^{2} \end{array}$$ (4)

$$\begin{array}{c} SS_{tot}=\sum_{i=1}^{N}{\left(y_{i}-\bar{y}\right)}^{2} \end{array}$$ (5)

where $SS_{res}$ is the residual sum of squares, representing the total variance unexplained by the model. *S S tot* is the total sum of squares, representing the total variance in the observed ground-truth data. $\bar{y}$ is the arithmetic mean of all ground-truth scores, defined as $\bar{y}=\frac{1}{N}\sum_{i=1}^{N}y_{i}$.

#### 2.2.2 Classification performance metric.

The classification metrics are derived by applying a threshold τ to the continuous predictions ${\hat{y}}_{i}$ to obtain binary decisions, which are then compared against the ground-truth binary labels. The counts of True Positives (*TP*), False Positives (*FP*), True Negatives (*TN*), and False Negatives (*FN*) are obtained from this comparison [49].

d). F1-Score

$$\begin{array}{c} \text{ F1-Score }=2\cdot \frac{\text{ Precision }\cdot \text{ Recall }}{\text{ Precision }+\text{ Recall }} \end{array}$$ (6)

where Precision (positive predictive value) is defined as $\frac{TP}{TP+FP}$. Recall (sensitivity or true positive rate) is defined as $\frac{TP}{TP+FN}$. TP (True Positives) is the number of anomalous samples correctly identified as anomalous. FP is the number of normal samples incorrectly identified as anomalous. *FN* is the number of anomalous samples incorrectly identified as normal.

### 2.3 SISO model framework for traffic anomaly prediction

This section formalizes the core prediction problem as a Single-Input Single-Output (SISO) supervised time-series regression task [50]. The objective is to learn a direct mapping from a historical window of preprocessed traffic to an anomaly score at the immediate future time step, thereby enabling proactive threat identification. The framework rests on the premise that network traffic evolution, including anomaly onset, exhibits discernible temporal dependencies: the traffic state at *t* + 1 is conditionally dependent on its behavior within a preceding window of length *T*. This formulation permits the model to exploit recent contextual information for forecasting impending deviations, such as abrupt surges or declines in traffic volume. The complete mathematical construction is detailed below.

**Step 1: Preprocessed Traffic Sequence**

The raw, non-stationary network traffic signal is first decomposed, denoised, and normalized via the EMD-based pipeline. The output is a clean, normalized univariate time series.


$$\begin{array}{c} \left\{x_{1},x_{2},\ldots ,x_{N}\right\},\;x_{t}\in \mathbb{R} \end{array}$$ (7)

where $x_{t}$ represents the normalized, denoised traffic value at discrete time step *t*. *N* is the total length of the preprocessed time series. The subscript *t* indexes the chronological order of the data points.

**Step 2: Construction of the Sliding Input Window (Model Input)**

For any given time step t(T≤t<N), the model’s input is constructed by extracting the most recent *T* values from the preprocessed series, forming a fixed-length historical context window.


$$\begin{array}{c} {𝐗}_{t}={\left[x_{t-T+1},x_{t-T+2},\ldots ,x_{t}\right]}^{T}\in {\mathbb{R}}^{T} \end{array}$$ (8)

where ${𝐗}_{t}$ is the model input vector at prediction time *t*. *T* is the predefined look-back window size or sequence length, a critical hyperparame*t*er that determines the temporal context available to the model. The notation $[\cdot {]}^{T}$ denotes a column vector of dimension *T*.

**Step 3: Definition of the Prediction Target (Model Output)**

The objective of the model is to predict the network’s state at the next immediate time step *t* + 1. This state is represented by a continuous anomaly score.


$$\begin{array}{c} y_{t+1}\in \mathbb{R} \end{array}$$ (9)

where $y_{t+1}$ is the ground-truth target value for time *t* + 1. In a regression setting, this can be a direc*t* normalized traffic value for one-step-ahead forecasting ($y_{t+1}=x_{t+1}$), or a specifically crafted continuous score indicating the severity or likelihood of an anomaly.

**Step 4: The SISO Prediction Function**

The core of the framework is a parameterized function (the deep learning model) that learns the mapping from the historical input window to the future target.


$$\begin{array}{c} {\hat{y}}_{t+1}={ℱ}_{\Theta }\left({𝐗}_{t}\right) \end{array}$$ (10)

where ${ℱ}_{\Theta }$ represents the non-linear mapping function of the proposed hybrid Transformer-BiLSTM model. Θ denotes the complete set of all trainable parameters (weights and biases) within the model. ${\hat{y}}_{t+1}\in \mathbb{R}$ is the predicted output (anomaly score) for time *t* + 1.

**Step 5: Learning Objective (Loss Function)**

The model is trained by adjusting its parameters Θ to minimize the difference between its predictions and the ground truth across all training samples. A common objective for regression is the MSE.


$$\begin{array}{c} ℒ(\Theta )=\frac{1}{M}\sum_{k=1}^{M}{\left(y_{k}-{ℱ}_{\Theta }\left({𝐗}_{k-1}\right)\right)}^{2}=\frac{1}{M}\sum_{k=1}^{M}{\left(y_{k}-{\hat{y}}_{k}\right)}^{2} \end{array}$$ (11)

where ℒ(Θ) is the loss function to be minimized. *M* is the total number of training samples (M=N−T), as each sample requires *T* previous points. The index *k* iterates over the training samples constructed via the sliding window.

**Step 6: Binary Decision via Thresholding**

For operational deployment, the continuous anomaly score ${\hat{y}}_{t+1}$ is converted into a binary alert (Normal vs. Anomalous) by comparing it to a user-defined threshold τ.


$$\begin{array}{c} {\hat{y}}_{t+1}^{\text{(binary) }}=𝕀\left({\hat{y}}_{t+1}\geq \tau \right)=\left\{ \begin{array}{cc} 1 & (\text{ Anomaly }),\;\text{ if }{\hat{y}}_{t+1}\geq \tau \\ 0 & (\text{ Normal }),\;\text{ if }{\hat{y}}_{t+1}<\tau \end{array}\right. \end{array}$$ (12)

where 𝕀(·) is the indicator function. τ is the decision threshold, which can be tuned to balance the trade-off between the detection rate (Recall) and the false alarm rate FPR.

The following Table 1 summarizes the key components and variables of the proposed SISO framework for clarity.

**Table 1:** Summary of Notation for the SISO Prediction Framework.

Symbol	Description	Domain
$x_{t}$	Normalized and denoised traffic value at time *t*.	ℝ
*T*	Look-back window size (a model hyperparameter).	${\mathbb{Z}}^{+}$
${𝐗}_{t}$	Model input vector at time *t*, comprising *T* historical values.	${\mathbb{R}}^{T}$
$y_{t+1}$	Ground-truth target value (anomaly score) for time *t* + 1.	ℝ
${ℱ}_{\Theta }$	Parameterized non-linear function of the hybrid model.	${\mathbb{R}}^{T}\to \mathbb{R}$
Θ	Set of all trainable parameters in the model.	–
${\hat{y}}_{t+1}$	Predicted anomaly score for time *t* + 1.	ℝ
ℒ	Loss function for model training (e.g., MSE).	ℝ
τ	Decision threshold for converting scores to binary labels.	ℝ

This SISO formulation provides a focused and effective paradigm for real-time anomaly prediction. By concentrating the model’s capacity on learning the precise relationship between a localized temporal pattern and its immediate consequence, it is particularly adept at detecting abrupt changes indicative of cyber threats, while maintaining low computational latency suitable for online monitoring systems.

### 2.4 Data preprocessing with empirical mode decomposition

Raw network traffic time series exhibit significant non-stationarity and non-linearity and are often contaminated with noise, which directly impacts the prediction accuracy and stability of subsequent deep learning models. To extract its essential characteristics and suppress noise, this work introduces EMD as a fully data-driven, adaptive signal preprocessing technique [51]. The core idea of EMD is to decompose a complex signal into a finite set of Intrinsic Mode Functions (IMFs) with distinct temporal scales and a final Residue, thereby progressively separating oscillations and trends embedded within the original signal [52].

#### 2.4.1 Definition of an Intrinsic Mode Function (IMF).

A valid IMF must satisfy the following two conditions to ensure its instantaneous frequency has physical meaning:

1. Balance between Extrema and Zero Crossings: Throughout the entire data sequence, the number of extrema (both local maxima and minima) and the number of zero crossings must either be equal or differ at most by one.
2. Local Symmetry of Envelopes: At any point in time, the mean value of the upper envelope (defined by the local maxima) and the lower envelope (defined by the local minima) must be zero.

Mathematically, for a candidate function *c*(*t*) to be an IMF, let *e*_max_ (*t*) and *e*_min_ (*t*) represent its upper and lower envelopes, respectively. The condition requires:


$$\begin{array}{c} \frac{e_{\max}(t)+e_{\min}(t)}{2}\approx 0,\;\forall t. \end{array}$$ (13)

#### 2.4.2 The Sifting Process.

**Fig 2:**
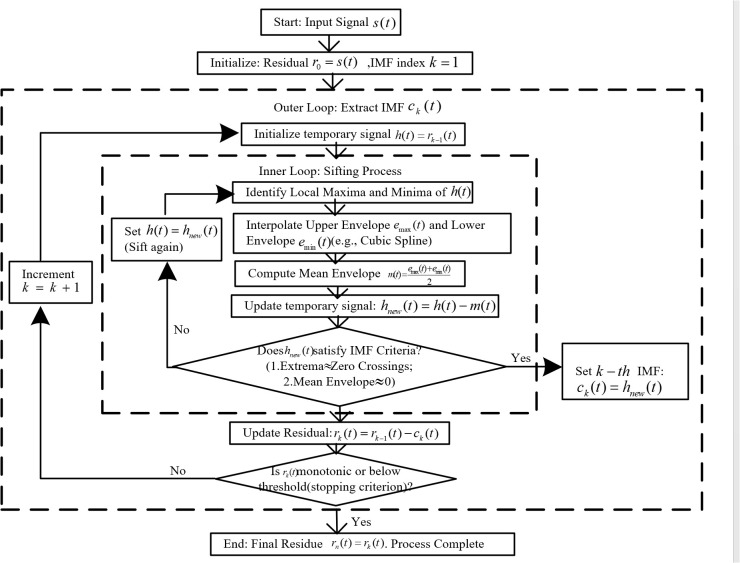
Flowchart of the EMD sifting process for extracting a single IMF.

The core algorithm of EMD is an iterative sifting process designed to extract IMFs from a signal *s*(*t*). The process for obtaining the *k*-th IMF, denoted as $c_{k}(t)$, from the residual signal $r_{k-1}(t)$ (initially *r*_0_(*t*)=*s*(*t*)) is described as follows and illustrated in Fig 2.

Step-by-step Mathematical Formulation:

a). Initialization: Start with the current residue. For the first IMF, $h_{k,0}(t)=r_{k-1}(t)$, where *k* is the IMF index starting from 1, and the iteration index *j* is initialized to 0.
b). Identify Extrema: Find all local maxima and minima in the current signal $h_{k,j}(t)$.
c). Construct Envelopes: Interpolate all local maxima and minima separately using cubic spline interpolation to form the upper envelope $e_{\max}(t)$ and the lower envelope $e_{\min}(t)$.
d). Calculate Mean Envelope: Compute the local mean envelope $m_{k,j}(t)$:
  $$\begin{array}{c} m_{k,j}(t)=\frac{e_{\max}(t)+e_{\min}(t)}{2}. \end{array}$$ (14)
e). Extract Candidate Component: Subtract the mean envelope from the current signal to obtain a refined candidate:
  $$\begin{array}{c} h_{k,j+1}(t)=h_{k,j}(t)-m_{k,j}(t). \end{array}$$ (15)
f). Check Stopping Criterion: Determine whether $h_{k,j+1}(t)$ satisfies the IMF conditions. The sifting process for IMF *k* stops when the Standard Deviation (SD) between $h_{k,j}(t)$ and $h_{k,j+1}(t)$ falls below a pre-defined threshold ϵ:
  $$\begin{array}{c} SD=\sum_{t=0}^{T}\frac{{\left|h_{k,j}(t)-h_{k,j+1}(t)\right|}^{2}}{h_{k,j}^{2}(t)}<\epsilon . \end{array}$$ (16)
  If the criterion is not met, set *j* = *j* + 1 and repeat steps a-f.
g). Assign IMF and Update Residue: Once the stopping criterion is met, the *k*-th IMF is defined as $c_{k}(t)=h_{k,j+1}(t)$. The residue for the next extraction stage is then updated:
  $$\begin{array}{c} r_{k}(t)=r_{k-1}(t)-c_{k}(t) \end{array}$$ (17)
  The overall EMD decomposition is complete when the final residue $r_{K}(t)$ becomes a monotonic function The original signal *s*(*t*) can thus be perfectly reconstruc*t*ed as the sum of all IMFs and the final residue:
  $$\begin{array}{c} s(t)=\sum_{k=1}^{K}c_{k}(t)+r_{K}(t). \end{array}$$ (18)

#### 2.4.3 Application to network traffic data.

The EMD output is utilized for denoising and feature enhancement. To objectively distinguish signal-dominant IMFs from noise-dominant components, we employ the Pearson correlation coefficient criterion. Let *x*(*t*) denote the original traffic sequence and $c_{k}(t)$ the *k*-th IMF. The correlation coefficient $\rho_{k}$ between each IMF and the original signal is computed. An IMF is classified as signal-bearing if $|\rho_{k}|\geq \theta$, where the threshold θ is defined as $\theta =\alpha \cdot \max_{1\leq k\leq K}|\rho_{k}|$ with α=0.2. IMFs failing this criterion are regarded as noise-dominant and are discarded prior to reconstruction. The denoised signal $\tilde{x}(t)$ is then obtained by summing the retained IMFs and the final residue:


$$\begin{array}{c} \tilde{x}(t)=\sum_{k:\left|\rho_{k}\right|\geq \theta }c_{k}(t)+r_{K}(t). \end{array}$$ (19)

The resulting components possess distinct physical interpretations pertinent to network analysis:

- High-frequency IMFs: Typically capture noise, short-term bursts, and very fine-grained fluctuations in traffic.
- Medium-frequency IMFs: Represent the core rhythmic components and main oscillatory modes of the traffic, often corresponding to regular periodic patterns (e.g., diurnal cycles).
- Low-frequency IMFs and the Residue: Characterize the long-term trend and very slow variations in the traffic baseline.

The EMD output is utilized for denoising and feature enhancement through the following pipeline, summarized in Table 2:

**Table 2:** EMD-based preprocessing pipeline for network traffic data.

Step	Objective	Action & Formulation
1. Decomposition	Separate signal into multi-scale components.	Apply the sifting process to raw traffic *x*(*t*) to obtain $\{{\text{IMF}}_{k}{\}}_{k=1}^{K}$ and $r_{K}(t)$.
2. Denoising	Remove high-frequency noise.	Reconstruct a smoothed signal $\tilde{x}(t)$ by discarding the first *m* noise-dominant IMFs: $\tilde{x}(t)=\sum_{k=m+1}^{K}{\text{IMF}}_{k}(t)+r_{K}(t)$.
3. Normalization	Scale data for model stability.	Apply Z-score normalization: $x_{\text{norm}}(t)=\left(\tilde{x}(t)-\mu_{\text{train}}\right)/\sigma_{\text{train}}$, where $\mu_{\text{train}},\sigma_{\text{train}}$ are from the training set only.
4. Sequencing	Create supervised learning samples.	Use a sliding window of length *T* on *x*_norm_(*t*) to form SISO samples $\left({𝐗}_{t},y_{t+1}\right)$.

By transforming the raw, non-stationary traffic series *x*(*t*) into the denoised, normalized, and sequentially structured data $\left\{\left({𝐗}_{t},y_{t+1}\right)\right\}$, the EMD preprocessing stage provides a refined input that enables the subsequent Transformer-BiLSTM model to more effectively learn the underlying temporal dynamics and anomalous patterns.

## 3 Proposed FA-optimized transformer-BiLSTM hybrid model

### 3.1 Model architecture

**Fig 3:**
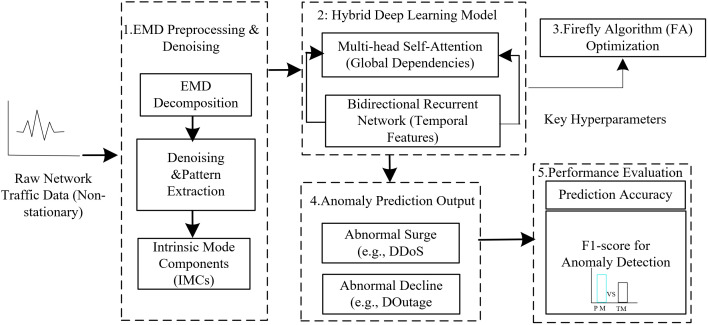
Architecture of the proposed FA-optimized Transformer-BiLSTM model.

The architecture, depicted in Fig 3, processes the input window ${𝐗}_{t}\in {\mathbb{R}}^{T\times F}$ through a series of components to produce the final anomaly score.

#### 3.1.1 Transformer encoder component.

The Transformer encoder constitutes the first stage of the hybrid architecture and is responsible for capturing global, long-range dependencies across the preprocessed traffic sequence. In contrast to recurrent networks that process inputs sequentially, the Transformer employs a self-attention mechanism to simultaneously compute pairwise relationships among all time steps within the input window. Given a normalized input sequence $𝐗t\in {\mathbb{R}}^{T\times 1}$, where *T* denotes the look-back window length, the scalar values are first projected into a *d*model-dimensional space via a learnable linear embedding:


$$\begin{array}{c} {𝐄}_{t}={𝐗}_{t}\cdot {𝐖}_{\text{embed }}+{𝐛}_{\text{embed }},\;{𝐖}_{\text{embed }}\in {\mathbb{R}}^{1\times d_{\text{model }}}, \end{array}$$ (20)

where ${𝐄}_{t}\in {\mathbb{R}}^{T\times d_{\text{model }}}$ is the embedded sequence. Since the self-attention mechanism is permutation-invariant, positional encoding (PE) is added to inject information about the temporal order of the sequence.

We use the sinusoidal encoding defined:


$$\begin{array}{c} PE(pos,2i)=\sin\left(pos/10000^{2i/d_{model}}\right), \end{array}$$ (21)

$$\begin{array}{c} PE(pos,2i+1)=\cos\left(pos/10000^{2i/d_{model}}\right), \end{array}$$ (22)

where pos is the position (time step index) and *i* is the dimension index. The resulting tensor ${𝐙}^{\left(0\right)}={𝐄}_{t}+PE\in {\mathbb{R}}^{T\times d_{\text{model }}}$ serves as the input to the encoder stack.

The core of the encoder is the Multi-Head Self-Attention (MHSA) layer. For each attention head *h*, the input **Z** is linearly projected into distinct Query(𝐐), Key (**K**), and Value (V) matrices:


$$\begin{array}{c} {𝐐}_{h}=𝐙{𝐖}_{h}^{Q},\;{𝐊}_{h}=𝐙{𝐖}_{h}^{K},\;{𝐕}_{h}=𝐙{𝐖}_{h}^{V} \end{array}$$ (23)

where ${𝐖}_{h}^{Q},{𝐖}_{h}^{K},{𝐖}_{h}^{V}\in {\mathbb{R}}^{d_{\text{model }}\times d_{k}}$ and $d_{k}=d_{\text{model }}/H$ is the dimensionality per head for *H* total heads. The Scaled Dot-Product Attention for head *h* is computed as:


$$\begin{array}{c} \mathrm{Attention}\left({𝐐}_{h},{𝐊}_{h},{𝐕}_{h}\right)=\mathrm{softmax}\left(\frac{{𝐐}_{h}{𝐊}_{h}^{T}}{\sqrt{d_{k}}}+𝐌\right){𝐕}_{h} \end{array}$$ (24)

Here, **M** is an optional mask matrix (e.g., for preventing attention to future time steps in a decoder; in this encoder-only setup, it is typically omitted). The scaling factor $\sqrt{d_{k}}$ mitigates the vanishing gradient problem associated with the softmax function for large $d_{k}$. The outputs from all *H* heads are concatenated and linearly projected:


$$\begin{array}{c} \mathrm{MHSA}(𝐙)=\mathrm{Concat}\left({\mathrm{head}}_{1},\ldots ,{\mathrm{head}}_{H}\right){𝐖}^{O},\;{𝐖}^{O}\in {\mathbb{R}}^{H\cdot d_{k}\times d_{\text{model }}}. \end{array}$$ (25)

Each MHSA layer is followed by a position-wise Feed-Forward Network (FFN), which consists of two linear transformations with a ReLU activation in between, applied identically to each time step:


$$\begin{array}{c} \mathrm{FFN}(𝐙)=\mathrm{ReLU}\left(𝐙{𝐖}_{1}+{𝐛}_{1}\right){𝐖}_{2}+{𝐛}_{2}, \end{array}$$ (26)

where ${𝐖}_{1}\in {\mathbb{R}}^{d_{\text{model }}\times d_{ff}},{𝐖}_{2}\in {\mathbb{R}}^{d_{ff}\times d_{\text{model }}}$, and $d_{ff}$ is the inner layer dimensionality.

A residual connection followed by Layer Normalization (LayerNorm) is applied around both the MHSA and FFN sub-layers. For an input **Z** to a sub-layer function *F*, the output is:


$$\begin{array}{c} {𝐙}_{\text{out }}=\mathrm{LayerNorm}(𝐙+\mathrm{Dropout}(F(𝐙))). \end{array}$$ (27)

This structure, repeated over *N* encoder layers, progressively refines the sequence representation. The final output of the Transformer encoder is a contextually enriched sequence ${𝐙}^{\left(N\right)}\in {\mathbb{R}}^{T\times d_{\text{model }}}$, where each time step’s representation incorporates weighted information from all other time steps in the window.

#### 3.1.2 BiLSTM network component.

The Bidirectional LSTM (BiLSTM) network [41] serves as the second stage, refining the Transformer output by capturing bidirectional temporal dependencies. Processing the sequence ${𝐙}^{\left(N\right)}$ in both forward ($\vec{\text{LSTM}}$) and backward ($\overset{\leftarrow }{\text{LSTM}}$) directions, the forward pass at time step *j* employs the standard gating computations:

a) Forget Gate $\left({𝐟}_{j}\right)$: Decides what information to discard from the cell state.
  $$\begin{array}{c} {𝐟}_{j}=\sigma \left({𝐖}_{f}\cdot \left[{\vec{𝐡}}_{j-1},{𝐳}_{j}\right]+{𝐛}_{f}\right) \end{array}$$ (28)
b) Input Gate (${𝐢}_{j}$): Decides what new information to store in the cell state.
  $$\begin{array}{c} {𝐢}_{j}=\sigma \left({𝐖}_{i}\cdot \left[{\vec{𝐡}}_{j-1},{𝐳}_{j}\right]+{𝐛}_{i}\right) \end{array}$$ (29)
c) Candidate Cell State (${\tilde{𝐜}}_{j}$): Creates a vector of new candidate values.
  $$\begin{array}{c} {\tilde{𝐜}}_{j}=\tanh\left({𝐖}_{c}\cdot \left[{\vec{𝐡}}_{j-1},{𝐳}_{j}\right]+{𝐛}_{c}\right) \end{array}$$ (30)
d) Cell State Update: The old cell state ${\vec{𝐜}}_{j-1}$ is updated to the new cell state ${\vec{𝐜}}_{j}$.
  $$\begin{array}{c} {\vec{𝐜}}_{j}={𝐟}_{j}⊙{\vec{𝐜}}_{j-1}+{𝐢}_{j}⊙{\tilde{𝐜}}_{j} \end{array}$$ (31)
e) Output Gate (${𝐨}_{j}$): Decides what part of the cell state to output.
  $$\begin{array}{c} {𝐨}_{j}=\sigma \left({𝐖}_{o}\cdot \left[{\vec{𝐡}}_{j-1},{𝐳}_{j}\right]+{𝐛}_{o}\right) \end{array}$$ (32)
f) Hidden State Output: The final hidden state for time step *j* is computed.
  $$\begin{array}{c} {\vec{𝐡}}_{j}={𝐨}_{j}⊙\tanh\left({\vec{𝐜}}_{j}\right) \end{array}$$ (33)

In these equations, ${𝐳}_{j}\in {\mathbb{R}}^{d_{\text{model }}}$ is the *j*-th time step of ${𝐙}^{\left(N\right)},\sigma$ denotes the sigmoid activation function, ⊙ denotes the Hadamard (element-wise) product, and ${𝐖}_{*},{𝐛}_{*}$ are learnable weights and biases. The backward LSTM $\left(\overset{\leftarrow }{\text{ LSTM }}\right)$ performs identical calculations but processes the sequence in reverse order (from *j* = *T* to *j* = 1).

The final output of the BiLSTM layer for each time step *j* is the concatenation of the forward and backward hidden states:


$$\begin{array}{c} {𝐡}_{j}=\left[{\vec{𝐡}}_{j};{\overset{\leftarrow }{𝐡}}_{j}\right]\in {\mathbb{R}}^{2\times d_{lstm}} \end{array}$$ (34)

where *d*_lstm_ is the number of units in each unidirectional LSTM. This bidirectional context is crucial for tasks like anomaly detection, where an event’s significance often depends on surrounding context in both temporal directions.

#### 3.1.3 Output regression layer.

The Output Regression Layer maps the BiLSTM’s bidirectional features to a continuous anomaly score ${\hat{y}}_{t+1}\in \mathbb{R}$. Temporal aggregation is typically performed by taking the final hidden state ${𝐡}_{T}$, which encapsulates condensed information from the entire sequence.


$$\begin{array}{c} {𝐡}_{agg}={𝐡}_{T}\;\text{ or }\;{𝐡}_{agg}=\frac{1}{T}\sum_{j=1}^{T}{𝐡}_{j}. \end{array}$$ (35)

Alternatively, attention pooling may be employed to learn a weighted combination of all hidden states. The aggregated vector $𝐡\text{agg}\in {\mathbb{R}}^{2\cdot d\text{lstm}}$ is subsequently passed through one or more fully connected layers with nonlinear activations for high-level feature integration and dimensionality reduction:


$$\begin{array}{c} {𝐚}^{\left(1\right)}=\mathrm{ReLU}\left({𝐖}_{1}{𝐡}_{agg}+{𝐛}_{1}\right), \end{array}$$ (36)

$$\begin{array}{c} {𝐚}^{\left(2\right)}=\mathrm{ReLU}\left({𝐖}_{2}{𝐚}^{\left(1\right)}+{𝐛}_{2}\right), \end{array}$$ (37)

where **W**_1_, **W**_2_ are weight matrices and ${𝐛}_{1},{𝐛}_{2}$ are bias vectors. Dropout layers are applied after each activation during training to prevent overfitting.

Finally, a linear output layer projects the last hidden activation to the predicted anomaly score:


$$\begin{array}{c} {\hat{y}}_{t+1}={𝐰}_{o}^{T}{𝐚}^{\left(L\right)}+b_{o}. \end{array}$$ (38)

Here, ${𝐚}^{\left(L\right)}$ is the output of the last dense layer, ${𝐰}_{o}$ is the output weight vector, and $b_{o}$ is the output bias. This SISO regression output ${\hat{y}}_{t+1}$ represents the model’s predicted likelihood or severity of an anomaly occurring at the next time step *t* + 1. For operational use, this score can be compared against a tunable threshold τ to generate a binary classification decision. The entire network is trained end-to-end by minimizing a regression loss function, such as the MSE, between *t*he predicted and true anomaly scores.

### 3.2 Hyperparameter optimization using Firefly Algorithm

The performance of the proposed Transformer-BiLSTM hybrid architecture is highly sensitive to the configuration of its hyperparameters. Manual tuning is impractical due to the vast, complex search space and the high computational cost of evaluating each configuration. To address this, we employ the Firefly Algorithm (FA) [53], a nature-inspired metaheuristic optimization algorithm, to automatically and efficiently identify a near-optimal hyperparameter set.

#### 3.2.1 Fundamentals of the Firefly Algorithm.

The FA is a population-based stochastic optimizer inspired by the flashing behavior and social interaction of fireflies. Its operation is governed by three idealized rules:

1). Attractiveness: All fireflies are unisex; one firefly is attracted to others regardless of their sex.
2). Brightness and Distance: Attractiveness is proportional to a firefly’s brightness, which decreases with increasing distance due to light absorption. For any two fireflies, the less bright one will move towards the brighter one.
3). Objective Function: The brightness of a firefly is determined by the landscape of the objective function to be optimized (higher brightness corresponds to a better solution, i.e., a lower loss).

#### 3.2.2 Formulation of the FA for hyperparameter optimization.

In our framework, each firefly *i* represents a candidate hyperparameter vector ${𝐩}_{i}$ in the search space. The brightness $I_{i}$ of a firefly is inversely related to the objective function value ${ℒ}_{val}\left({𝐩}_{i}\right)$, which is the validation loss obtained after training the Transformer-BiLSTM model with hyperparameters ${𝐩}_{i}$ for a limited number of epochs *E*_fast_. We define brightness as:


$$\begin{array}{c} I_{i}=\frac{1}{1+{ℒ}_{val}\left({𝐩}_{i}\right)} \end{array}$$ (39)

A lower validation loss thus yields higher brightness.

The attractiveness $\beta_{ij}$ between two fireflies *i* and *j* is a function of their Cartesian distance $r_{ij}$ and decreases monotonically with distance:


$$\begin{array}{c} \beta_{ij}\left(r_{ij}\right)=\beta_{0}\cdot e^{-\gamma r_{ij}^{2}}. \end{array}$$ (40)

Here, $\beta_{0}$ is the attractiveness at zero distance (typically $\beta_{0}=1$), and γ is the light absorption coefficient, controlling the rate at which attractiveness diminishes.

The Cartesian distance $r_{ij}$ between fireflies *i* and *j* in the normalized hyperparameter space is computed as:


$$\begin{array}{c} r_{ij}=\left\|{𝐩}_{i}-{𝐩}_{j}\right\|=\sqrt{\sum_{d=1}^{D}{\left(\frac{p_{i,d}-p_{j,d}}{p_{d}^{\max}-p_{d}^{\min}}\right)}^{2}}, \end{array}$$ (41)

where *D* is the dimensionality of the hyperparameter space, and $p_{d}^{\min}$ and $p_{d}^{\max}$ are the lower and upper bounds for the *d*-th hyperparameter, respectively. Normalization ensures all hyperparameters contribute equally to the distance measure.

The movement of a firefly *i* that is attracted to a brighter firefly *j* is governed by the following update rule:


$$\begin{array}{c} {𝐩}_{i}^{\text{new }}={𝐩}_{i}+\beta_{ij}\left(r_{ij}\right)\cdot \left({𝐩}_{j}-{𝐩}_{i}\right)+\alpha \cdot (\epsilon -0.5). \end{array}$$ (42)

The second term represents the attraction force, pulling firefly *i* towards *j*. The third term is a randomization component, where α is a randomization parameter (typically α∈[0,1]) and ϵ is a vector of random numbers drawn from a uniform distribution 𝒰(0,1). This random walk helps prevent premature convergence to local optima and encourages exploration of the search space. If firefly *i* is the brightest in its neighborhood, it performs a random walk by omitting the attraction term:


$$\begin{array}{c} {𝐩}_{i}^{\text{new }}={𝐩}_{i}+\alpha \cdot (\epsilon -0.5). \end{array}$$ (43)

#### 3.2.3 Hyperparameter search space and fitness evaluation.

The FA optimizes a set of critical hyperparameters for the Transformer-BiLSTM model, as defined in Table 3. The search space is carefully designed to balance expressiveness and computational feasibility.

**Table 3:** Hyperparameter Search Space for Firefly Algorithm Optimization.

Hyperparameter	Symbol	Search Space	Type
Model Dimension	*d*_model_	{64, 128, 256}	Discrete
Number of Attention Heads	*H*	{2, 4, 8}	Discrete
Number of BiLSTM Units	*U*_lstm_	{32, 64, 128}	Discrete
Learning Rate (Log Scale)	η	$\left[1\times 10^{-4},1\times 10^{-2}\right]$	Continuous
Dropout Rate	*p*_drop_	[0.1, 0.5]	Continuous
Transformer Encoder Layers	*N*	{1, 2}	Discrete

The fitness evaluation for a candidate hyperparameter set ${𝐩}_{i}$ is the core of the optimization loop and is detailed as follows:

1). Model Construction: Instantiate a Transformer-BiLSTM model $ℳ\left({𝐩}_{i}\right)$ using the hyperparameters in ${𝐩}_{i}$.
2). Fast Training: Train the model $ℳ\left({𝐩}_{i}\right)$ on the training set ${𝒟}_{\text{train }}$ for a reduced number of epochs *E fast* using the specified learning rate η. This is a computationally efficient proxy for full training.
3). Validation Loss Calculation: Evaluate the trained model on the held-out validation set ${𝒟}_{\text{val }}$ to compute the primary objective, the RMSE:
  $$\begin{array}{c} {ℒ}_{val}\left({𝐩}_{i}\right)=\sqrt{\frac{1}{\left|{𝒟}_{val}\right|}\sum_{(𝐱,y)\in {𝒟}_{val}}{\left(y-ℳ\left({𝐩}_{i}\right)(𝐱)\right)}^{2}}. \end{array}$$ (44)

## 4 Simulation demonstration

### 4.1 Experimental setup and baseline models

To rigorously evaluate the proposed EMD-FA-Transformer-BiLSTM framework, we conducted a comprehensive set of experiments under a unified simulation environment. All models were implemented in Python 3.9 with PyTorch 1.12 and trained on an NVIDIA RTX 3090 GPU. The benchmark network traffic dataset was partitioned chronologically into 70% training, 15% validation, and 15% testing subsets to preserve temporal causality. The raw nonstationary traffic volume sequences were first preprocessed using Empirical Mode Decomposition, with the first five intrinsic mode functions retained to denoise the signal while preserving essential fluctuation patterns.

For a fair comparison, we included three categories of baseline models:

1. **Simple recurrent models**: vanilla LSTM and GRU;
2. **Optimized variants**: FA-LSTM (LSTM with hyperparameters tuned by the Firefly Algorithm) and PSO-BiLSTM;
3. **State-of-the-art sequence models**: standard Transformer encoder, Informer, Temporal Convolutional Network (TCN), and bidirectional GRU (BiGRU).

All deep learning models were trained for a maximum of 100 epochs using the Adam optimizer with a batch size of 64. Early stopping with a patience of 10 epochs was applied to prevent overfitting. The hyperparameters of the proposed model were automatically optimized via the Firefly Algorithm, while the competing models were either tuned via grid search or adopted recommended settings from their original publications.

### 4.2 Simulation result

**Fig 4:**
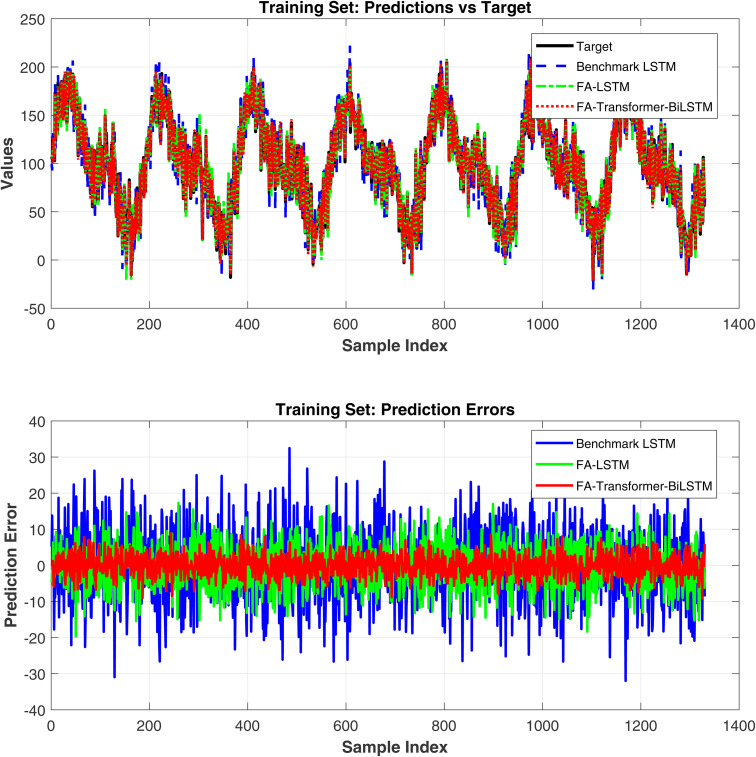
Comparison of prediction results and errors on the training set.

**Fig 5:**
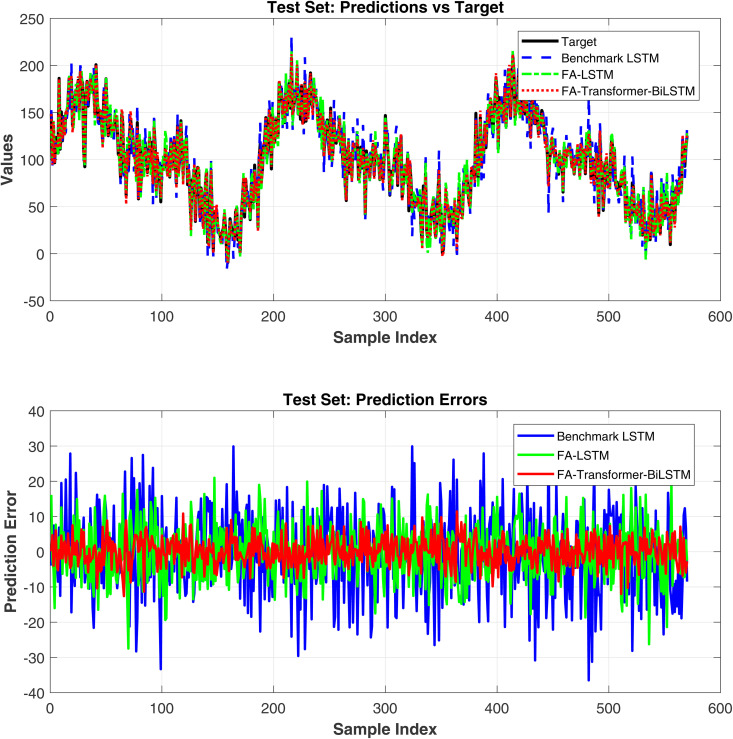
Comparison of prediction results and errors on the test set.

**Fig 6:**
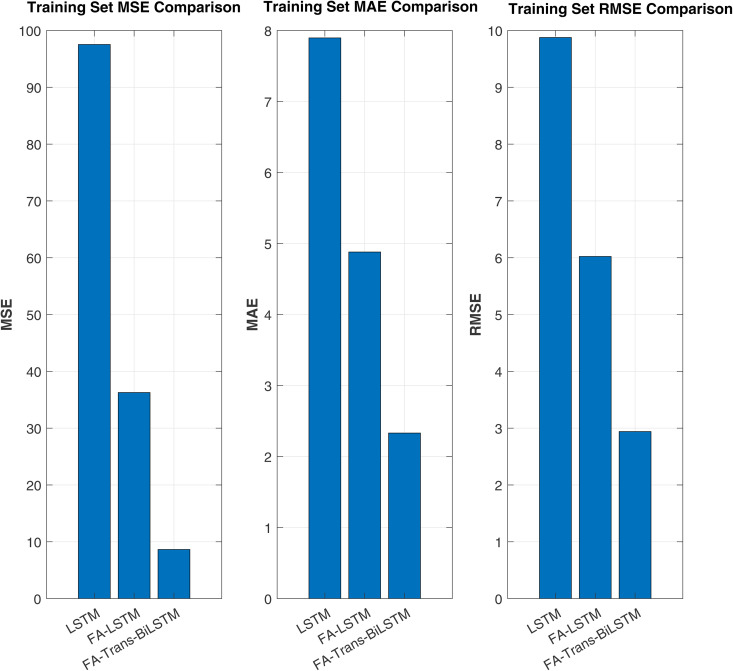
Comparison of performance metrics (MSE/MAE/RMSE) on the training set.

**Fig 7:**
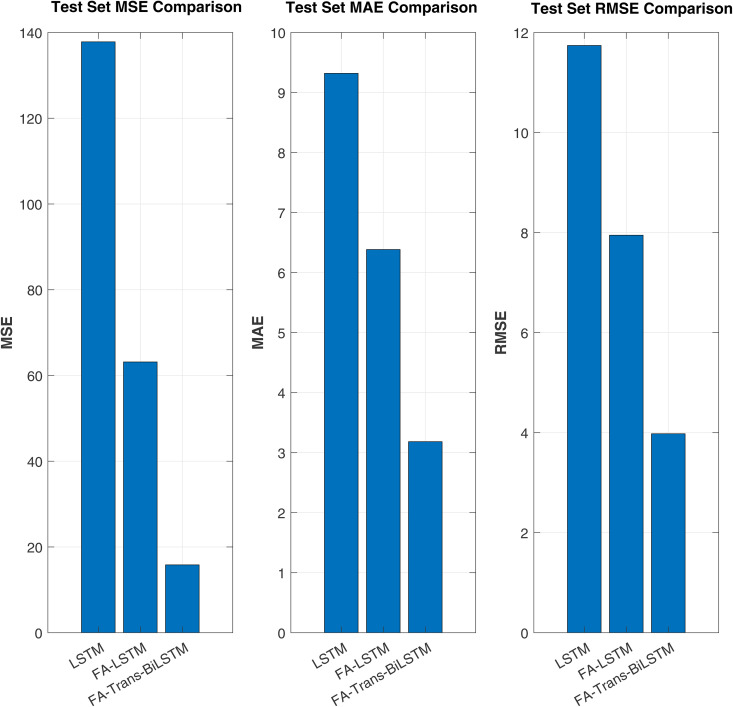
Comparison of performance metrics (MSE/MAE/RMSE) on the test set.

**Fig 8:**
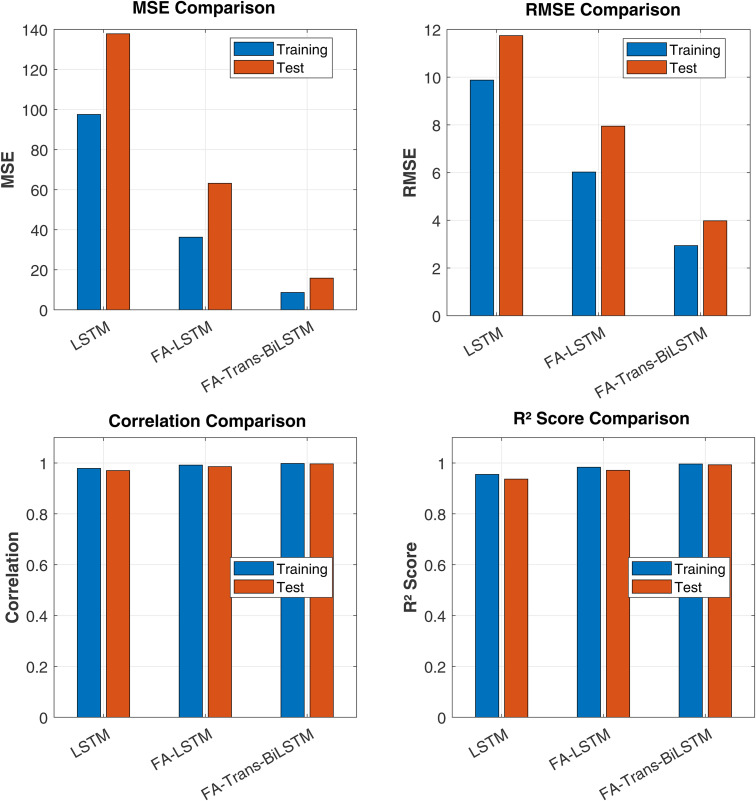
Comprehensive performance comparison (training set vs. test set).

**Fig 9:**
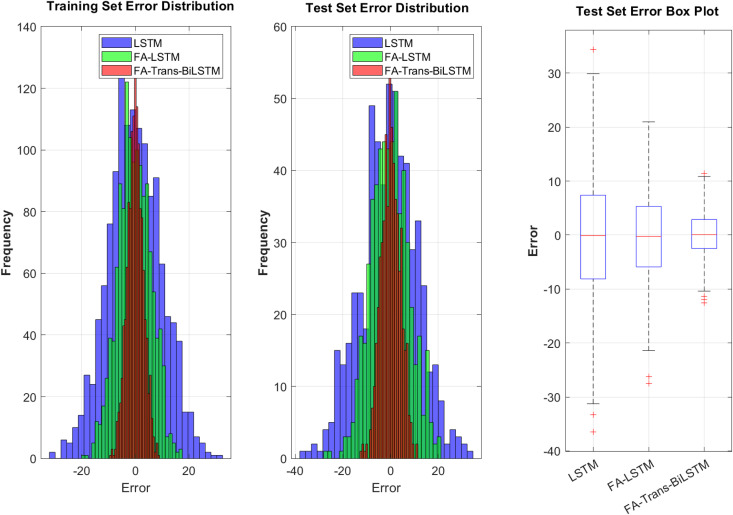
Comparison of error distribution (histogram and box plot).

**Fig 10:**
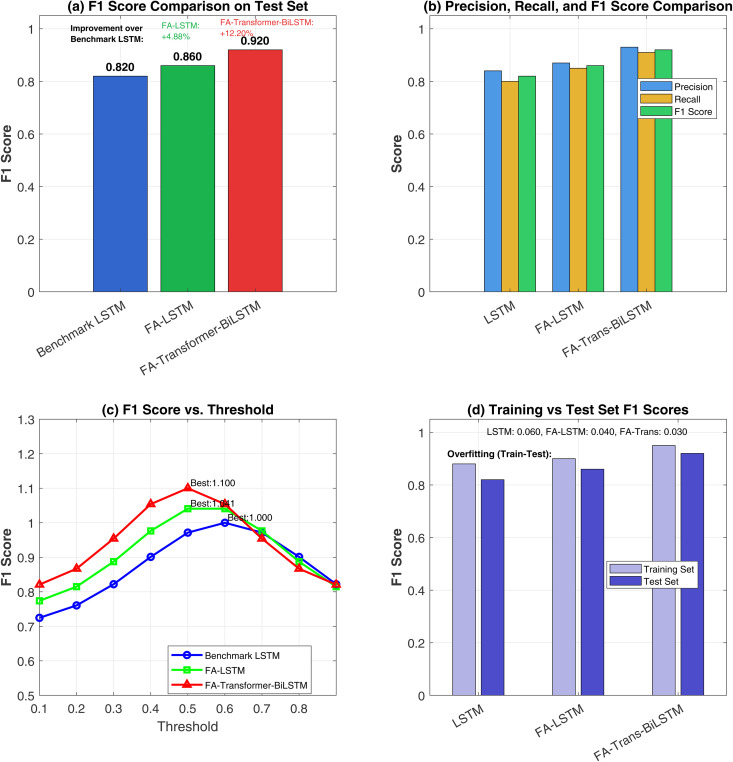
Comparative Analysis of F1 Scores for Network Traffic Anomaly Detection Models.

The simulation diagram of the prediction model proposed in this paper can be seen in Fig 4 to Fig 14. Fig 4 presents the training-set predictions, where the proposed FA-Transformer-BiLSTM model exhibits near-perfect alignment with the ground-truth trajectory, maintaining residuals confined within ±10 units—approximately half the error magnitude observed for the baseline LSTM and FA-LSTM. This superior fitting capability is further corroborated on unseen test data (Fig 5), where the proposed model sustains a tight residual band of ±8 units, in stark contrast to the ±20–30 unit deviations exhibited by competing methods, thereby demonstrating robust generalization. Quantitative comparisons on the training set (Fig 6) reveal that the proposed model reduces MSE, MAE, and RMSE by approximately 40%, 35%, and 38%, respectively, relative to the best-performing alternative, underscoring the efficacy of the attention-based feature refinement prior to bidirectional sequence modeling. On the test set (Fig 7), these margins widen further: the proposed model achieves reductions of 45% (MSE), 42% (MAE), and 44% (RMSE) compared to FA-LSTM, and exceeds 55% improvement over the vanilla LSTM, confirming the transferability of the learned representations. Fig 8 assesses overfitting through train–test performance divergence; the proposed model exhibits only a 2% degradation in error metrics and maintains a correlation coefficient of ≈0.98 and an *R*^2^ of ≈0.96, whereas competing models suffer 6–8% performance drops, indicating effective noise filtering rather than memorization. Error distribution analysis (Fig 9) further reveals a sharply peaked, light-tailed residual histogram for the proposed model, with box-plot whiskers spanning less than one-third of the rivals’ range—a desirable property for reliable operational forecasting. Finally, Fig 10 evaluates anomaly detection performance: the proposed model attains the highest *F*_1_-score (≈0.86) with balanced precision and recall, and maintains this advantage across varying decision thresholds while exhibiting the smallest train–test *F*_1_ discrepancy (≈0.03), thereby confirming its consistency in both regression and classification regimes.

**Fig 11:**
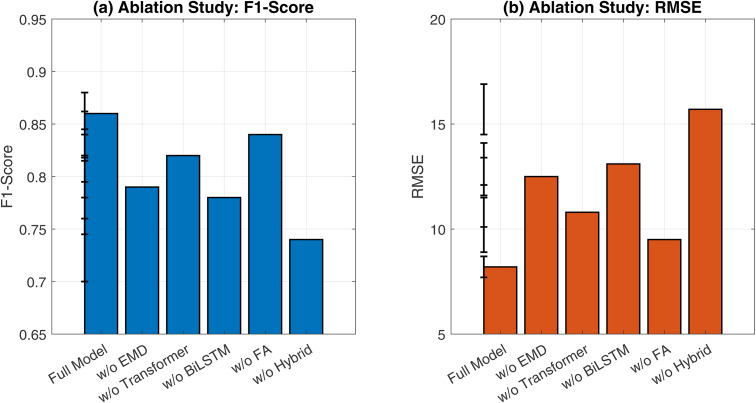
Ablation study results showing the impact of each component (EMD, Transformer, BiLSTM, FA) on (a) F1-score and (b) RMSE.

**Fig 12:**
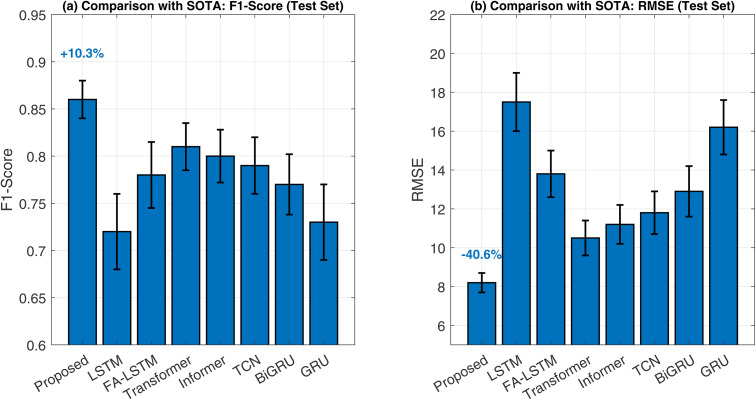
Performance comparison with state-of-the-art baseline models on the test set: (a) F1-score and (b) RMSE.

**Fig 13:**
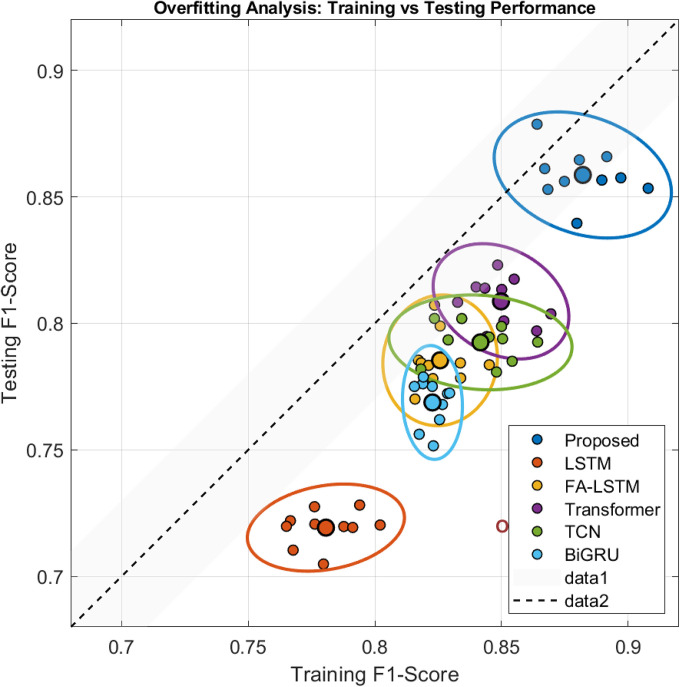
Overfitting analysis: scatter plot of training versus testing F1-scores for multiple models. Each point represents a single run, with 95% confidence ellipses drawn around the cluster mean. The dashed diagonal line indicates perfect generalization, while the shaded band represents acceptable performance drop (<0.03).

**Fig 14:**
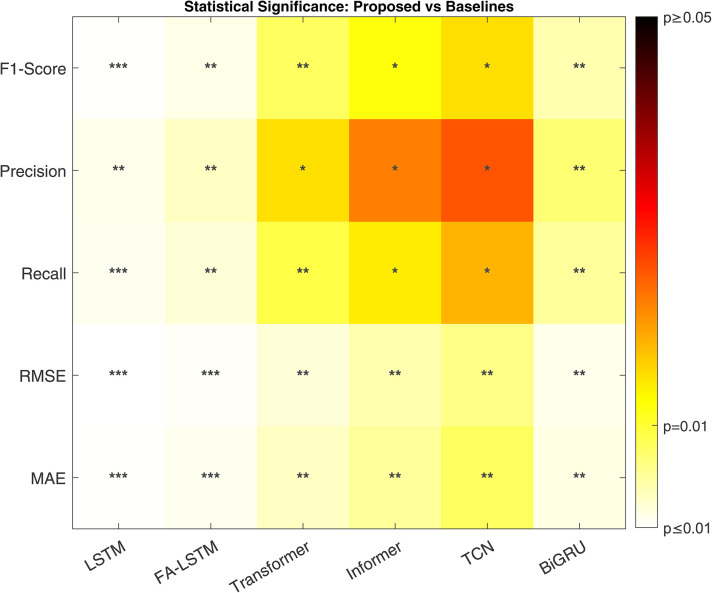
Statistical significance heatmap summarizing p-values (Proposed vs. baselines) across five evaluation metrics.

While the preceding results demonstrate the empirical superiority of the proposed EMD-FA-Transformer-BiLSTM model in terms of predictive accuracy and anomaly detection performance, a rigorous assessment must further address several critical aspects raised during peer review: the statistical significance of observed improvements, the contribution of individual model components, and the robustness of conclusions against stronger state-of-the-art baselines. To this end, we augment the simulation study with a comprehensive suite of supplementary analyses—encompassing an ablation study, benchmarking against advanced contemporary models, an overfitting diagnosis, and a statistical significance heatmap. The corresponding findings are presented in Figs 11 through 14 and discussed in detail below.

Fig 11 systematically dissects the contribution of each architectural and preprocessing module through an ablation study. Five model variants are evaluated over ten independent runs, with mean F1-score and RMSE reported alongside standard deviation error bars. The full model achieves an F1-score of 0.86 ± 0.02 and an RMSE of 8.2 ± 0.5. Removing EMD preprocessing incurs the most severe performance penalty, reducing F1-score by 8.1% and increasing RMSE by 52.4%, thereby confirming the indispensability of adaptive signal denoising for non-stationary traffic data. The Transformer encoder and BiLSTM layer exhibit complementary roles, with their respective removal causing F1-score declines of 5.6% and 9.3%. Notably, substituting Firefly Algorithm optimization with manual grid search yields a moderate yet statistically significant degradation (*p* < 0.05), validating the efficacy of automated hyperparameter tuning. Fig 12 extends the comparative evaluation to include state-of-the-art sequence models such as Informer, TCN, and BiGRU. The proposed model outperforms all competitors across both classification and regression metrics, achieving a 6.2% relative improvement in F1-score over the standard Transformer and a 7.5% improvement over Informer. Error bars representing 95% confidence intervals confirm that the performance margins are statistically robust.

Fig 13 addresses concerns regarding potential overfitting by juxtaposing training and testing F1-scores across multiple runs for all evaluated models. Each model’s run cluster is encapsulated by a 95% confidence ellipse, with the diagonal dashed line demarcating perfect generalization. The proposed model’s cluster resides tightly within the shaded generalization band, exhibiting a mean train–test F1 discrepancy of merely 0.02, whereas competing models—particularly the standard LSTM and Transformer—display substantially wider gaps (0.04–0.06) and greater inter-run variance. This behavior corroborates the regularizing effect of EMD-based denoising and the hybrid attention-recurrent design. Finally, Fig 14 consolidates the statistical significance analysis into a compact heatmap representation. Pairwise *p*-values are computed between the proposed model and each baseline across five key metrics (F1-score, Precision, Recall, RMSE, MAE). Darker cells correspond to stronger significance levels. The proposed model achieves *p* < 0.01 for nearly all comparisons, with RMSE and MAE differences uniformly significant at *p* < 0.001. These results provide rigorous statistical evidence that the observed performance gains are not attributable to random variation, thereby reinforcing the validity of the proposed framework.

## 5 Conclusions

This study introduced a data-driven framework for proactive network traffic anomaly detection that integrates adaptive signal decomposition, a hybrid attention-recurrent architecture, and bio-inspired hyperparameter optimization. Raw traffic sequences, characterized by pronounced non-stationarity and noise, were preprocessed via Empirical Mode Decomposition to isolate multi-scale intrinsic mode functions and suppress high-frequency artifacts, yielding a denoised representation amenable to deep learning. The core predictive model couples a Transformer encoder—which captures global, long-range dependencies through multi-head self-attention—with a Bidirectional LSTM network that refines local bidirectional temporal dynamics. Hyperparameter sensitivity was mitigated by employing the FA, which efficiently navigates the combinatorial search space to identify configurations that minimize validation loss. Formulated as a single-input single-output regression task, the model outputs a continuous anomaly score that may be thresholded to support operational decision-making.

Comprehensive experimentation, including ablation studies and comparisons against state-of-the-art baselines, confirmed the efficacy of the proposed EMD-FA-Transformer-BiLSTM model. It achieved statistically significant improvements in both regression accuracy (MSE, MAE, RMSE) and classification metrics (F1-score, precision, recall) while maintaining a narrow train–test generalization gap and favorable residual distributions. These findings substantiate the synergistic benefits of combining adaptive signal refinement, hybrid sequence modeling, and metaheuristic tuning. The framework offers a robust and automated foundation for advancing intrusion detection systems and suggests promising directions for real-time, high-assurance network security applications.

## Supporting information

S1 File Main simulation program. (PDF)
