# Supplementary material for: A hybrid transformer-BiLSTM model optimized with Firefly Algorithm for network traffic anomaly detection
Source: PLoS One. 2026 Jun 17;21(6):e0341920. doi: 10.1371/journal.pone.0341920 (PMC13274867; doi:10.1371/journal.pone.0341920)
Supplement: S1 File — (PDF) [file pone.0341920.s001.pdf]

```
%% ===========================================================================
% MAIN SIMULATION CODE FOR EMD-FA-OPTIMIZED HYBRID MODEL
% Network Traffic Anomaly Detection System - MATLAB Version
% ===========================================================================

clear; clc; close all;
addpath(genpath(pwd));

%% 1. SIMULATION PARAMETERS SETUP
fprintf('=========== NETWORK TRAFFIC ANOMALY DETECTION ===========\n');
fprintf('EMD-FA-OPTIMIZED TRANSFORMER-BILSTM HYBRID MODEL\n');
fprintf('=========================================================\n\n');

% Data parameters
dataParams.datasetName = 'CIC-IDS2017';
dataParams.trainRatio = 0.7;
dataParams.valRatio = 0.15;
dataParams.testRatio = 0.15;
dataParams.sequenceLength = 50;
dataParams.overlapRatio = 0.5;

% EMD parameters
emdParams.nIMFs = 5;                    % Number of Intrinsic Mode Functions
emdParams.maxIterations = 500;          % Maximum sifting iterations
emdParams.stopThresh = 0.05;            % Stopping threshold

% Firefly Algorithm parameters
faParams.nFireflies = 20;               % Population size
faParams.maxIterations = 50;            % Maximum iterations
faParams.alpha = 0.2;                   % Randomness coefficient
faParams.beta0 = 1.0;                   % Attractiveness base
faParams.gamma = 1.0;                   % Light absorption coefficient

% Model architecture parameters (initial values, will be optimized by FA)
modelParams.lstmHiddenUnits = [64, 128];          % Range for LSTM hidden units
modelParams.transformerHeads = [4, 8];            % Range for attention heads
modelParams.learningRate = [0.0001, 0.01];        % Range for learning rate
modelParams.dropoutRate = [0.1, 0.4];             % Range for dropout rate

% Training parameters
trainParams.maxEpochs = 100;
trainParams.miniBatchSize = 64;
trainParams.patience = 10;              % Early stopping patience
trainParams.optimizer = 'adam';         % 'adam' or 'sgd'
```

```
% Evaluation parameters
evalParams.threshold = 0.5;                % Classification threshold
evalParams.f1Beta = 1;                      % F1-score beta parameter (1 for F1)

%% 2. DATA LOADING AND PREPROCESSING WITH EMD
fprintf('[1] Loading and preprocessing data with EMD...\n');

% Load dataset (simulated data - replace with actual loading)
[rawData, labels] = loadSimulatedData(10000, 20); % 10000 samples, 20 features

% Apply EMD decomposition to each feature
fprintf('      Applying Empirical Mode Decomposition...\n');
[imfData, residualData] = applyEMDDecomposition(rawData, emdParams);

% Feature selection: use first nIMFs + residual
processedData = imfData(:, :, 1:emdParams.nIMFs);
processedData = cat(3, processedData, residualData);

% Reshape for sequence processing
[sequences, sequenceLabels] = createSequences(processedData, labels, ...
    dataParams.sequenceLength, dataParams.overlapRatio);

% Normalize sequences
[normalizedSequences, normalizationParams] = normalizeSequences(sequences);

% Split into train, validation, and test sets
[trainData, valData, testData, trainLabels, valLabels, testLabels] = ...
    splitDataset(normalizedSequences, sequenceLabels, ...
    dataParams.trainRatio, dataParams.valRatio, dataParams.testRatio);

fprintf('      Data statistics:\n');
fprintf('      - Total samples: %d\n', size(sequences, 4));
fprintf('      - Training set: %d samples\n', size(trainData, 4));
fprintf('      - Validation set: %d samples\n', size(valData, 4));
fprintf('      - Test set: %d samples\n', size(testData, 4));
fprintf('      - Sequence length: %d\n', dataParams.sequenceLength);
fprintf('      - Features per timestep: %d\n', size(trainData, 3));

%% 3. FIREFLY ALGORITHM FOR HYPERPARAMETER OPTIMIZATION
fprintf('\n[2] Optimizing hyperparameters with Firefly Algorithm...\n');

% Define search space for FA
searchSpace = struct();
```

```
searchSpace.lstmUnits = modelParams.lstmHiddenUnits;
searchSpace.attentionHeads = modelParams.transformerHeads;
searchSpace.learningRate = modelParams.learningRate;
searchSpace.dropoutRate = modelParams.dropoutRate;

% Run Firefly Algorithm optimization
fprintf('      Running FA optimization (%d fireflies, %d iterations)...\n', ...
        faParams.nFireflies, faParams.maxIterations);

[bestParams, bestFitness, fitnessHistory] = fireflyAlgorithmOptimization(...
        @modelFitnessFunction, searchSpace, faParams, ...
        trainData, trainLabels, valData, valLabels, trainParams);

fprintf('      Best parameters found:\n');
fprintf('      - LSTM hidden units: %d\n', bestParams.lstmUnits);
fprintf('      - Transformer heads: %d\n', bestParams.attentionHeads);
fprintf('      - Learning rate: %.6f\n', bestParams.learningRate);
fprintf('      - Dropout rate: %.3f\n', bestParams.dropoutRate);
fprintf('      - Best validation loss: %.6f\n', bestFitness);

% Plot FA convergence
figure('Position', [100, 100, 600, 400]);
plot(1:length(fitnessHistory), fitnessHistory, 'b-o', 'LineWidth', 2);
xlabel('Iteration', 'FontSize', 12, 'FontWeight', 'bold');
ylabel('Validation Loss', 'FontSize', 12, 'FontWeight', 'bold');
title('Firefly Algorithm Convergence', 'FontSize', 14, 'FontWeight', 'bold');
grid on;
box on;

%% 4. MODEL TRAINING WITH OPTIMIZED PARAMETERS
fprintf('\n[3] Training models with optimized parameters...\n');

% 4.1 Train Baseline LSTM (without FA optimization)
fprintf('      Training Baseline LSTM...\n');
baselineParams = struct();
baselineParams.lstmUnits = 128;
baselineParams.learningRate = 0.001;
baselineParams.dropoutRate = 0.2;

[baselineModel, baselineHistory] = trainLSTMModel(...
        trainData, trainLabels, valData, valLabels, baselineParams, trainParams);

% 4.2 Train FA-LSTM (LSTM with FA-optimized parameters)
fprintf('      Training FA-LSTM...\n');
```

```
faLSTMParams = struct();
faLSTMParams.lstmUnits = bestParams.lstmUnits;
faLSTMParams.learningRate = bestParams.learningRate;
faLSTMParams.dropoutRate = bestParams.dropoutRate;

[faLSTMModel, faLSTMHistory] = trainLSTMModel(...
    trainData, trainLabels, valData, valLabels, faLSTMParams, trainParams);

% 4.3 Train Proposed FA-Transformer-BiLSTM
fprintf('    Training FA-Transformer-BiLSTM...\n');
[faTransformerModel, faTransformerHistory] = trainTransformerBiLSTMModel(...
    trainData, trainLabels, valData, valLabels, bestParams, trainParams);

%% 5. MODEL EVALUATION AND F1-SCORE CALCULATION
fprintf('\n[4] Evaluating models on test set...\n');

% 5.1 Make predictions
fprintf('    Making predictions...\n');

% Baseline LSTM predictions
[baselinePreds, baselineScores] = predictLSTMModel(baselineModel, testData);
baselineBinaryPreds = baselineScores >= evalParams.threshold;

% FA-LSTM predictions
[faLSTMPreds, faLSTMScores] = predictLSTMModel(faLSTMModel, testData);
faLSTMBinaryPreds = faLSTMScores >= evalParams.threshold;

% FA-Transformer-BiLSTM predictions
[faTransformerPreds, faTransformerScores] = predictTransformerBiLSTMModel(...
    faTransformerModel, testData);
faTransformerBinaryPreds = faTransformerScores >= evalParams.threshold;

% 5.2 Calculate F1-scores and other metrics
fprintf('    Calculating evaluation metrics...\n');

% Baseline LSTM metrics
baselineMetrics = calculateClassificationMetrics(...
    testLabels, baselineBinaryPreds, baselineScores, evalParams);

% FA-LSTM metrics
faLSTMMetrics = calculateClassificationMetrics(...
    testLabels, faLSTMBinaryPreds, faLSTMScores, evalParams);

% FA-Transformer-BiLSTM metrics
```

```
faTransformerMetrics = calculateClassificationMetrics(...
        testLabels, faTransformerBinaryPreds, faTransformerScores, evalParams);

% 5.3 Display results
fprintf('\n============================================================\n');
fprintf('EVALUATION RESULTS\n');
fprintf('============================================================\n');

displayResults('Baseline LSTM', baselineMetrics);
displayResults('FA-LSTM', faLSTMMetrics);
displayResults('FA-Transformer-BiLSTM', faTransformerMetrics);

%% 6. VISUALIZATION OF RESULTS
fprintf('\n[5] Generating performance visualizations...\n');

% 6.1 Training history comparison
plotTrainingHistory(baselineHistory, faLSTMHistory, faTransformerHistory);

% 6.2 F1-score and metrics comparison
plotMetricsComparison(baselineMetrics, faLSTMMetrics, faTransformerMetrics);

% 6.3 Confusion matrices
plotConfusionMatrices(testLabels, ...
        baselineBinaryPreds, faLSTMBinaryPreds, faTransformerBinaryPreds);

% 6.4 ROC curves
plotROCCurves(testLabels, ...
        baselineScores, faLSTMScores, faTransformerScores);

% 6.5 Precision-Recall curves
plotPrecisionRecallCurves(testLabels, ...
        baselineScores, faLSTMScores, faTransformerScores);

% 6.6 Error analysis
plotErrorAnalysis(testData, testLabels, ...
        baselineBinaryPreds, faLSTMBinaryPreds, faTransformerBinaryPreds);

fprintf('\n============================================================\n');
fprintf('SIMULATION COMPLETED SUCCESSFULLY\n');
fprintf('============================================================\n');

%% ================================================================================
% SUPPORTING FUNCTIONS
% ================================================================================
```

```
function [data, labels] = loadSimulatedData(nSamples, nFeatures)
    % Generate simulated network traffic data
    % Replace this with actual data loading function

    % Normal traffic pattern (sinusoidal + noise)
    time = linspace(0, 10*pi, nSamples)';
    normalPattern = sin(time) + 0.5*sin(3*time) + 0.2*randn(nSamples, 1);

    % Anomaly patterns (spikes and drops)
    anomalyPattern = zeros(nSamples, 1);
    anomalyIndices = randperm(nSamples, floor(0.1*nSamples)); % 10% anomalies
    anomalyPattern(anomalyIndices) = 3*randn(length(anomalyIndices), 1);

    % Combine patterns
    mainSignal = normalPattern + anomalyPattern;

    % Create multiple features with correlations
    data = zeros(nSamples, nFeatures);
    data(:, 1) = mainSignal;

    for i = 2:nFeatures
        data(:, i) = 0.7*data(:, i-1) + 0.3*randn(nSamples, 1);
    end

    % Labels (1 for anomaly, 0 for normal)
    labels = double(anomalyPattern ~= 0);

    % Add some noise to make classification challenging
    labels = labels + 0.05*randn(size(labels));
    labels = min(max(labels, 0), 1); % Clip to [0, 1]
end

function [imfData, residualData] = applyEMDDecomposition(data, params)
    % Apply EMD to each feature channel
    [nSamples, nFeatures] = size(data);
    imfData = zeros(nSamples, nFeatures, params.nIMFs);
    residualData = zeros(nSamples, nFeatures);

    for f = 1:nFeatures
        signal = data(:, f);

        % Simplified EMD implementation (in practice, use proper EMD)
        % This is a placeholder - replace with actual EMD implementation
```

```
        for imf = 1:params.nIMFs
            % Extract IMF using simplified method
            frequency = 0.1 * imf;    % Decreasing frequency for higher IMFs
            imfData(:, f, imf) = 0.5 * sin(2*pi*frequency*(1:nSamples)') .* ...
                exp(-0.01*(1:nSamples)');
        end

        % Residual is the original minus sum of IMFs
        residualData(:, f) = signal - sum(imfData(:, f, :), 3);
    end
end

function [sequences, labels] = createSequences(data, labels, seqLength, overlap)
    % Create sequences from time series data
    [nSamples, nFeatures, nChannels] = size(data);
    stepSize = floor(seqLength * (1 - overlap));

    nSequences = floor((nSamples - seqLength) / stepSize) + 1;

    sequences = zeros(seqLength, nFeatures, nChannels, nSequences);
    sequenceLabels = zeros(nSequences, 1);

    for i = 1:nSequences
        startIdx = (i-1)*stepSize + 1;
        endIdx = startIdx + seqLength - 1;

        sequences(:, :, :, i) = data(startIdx:endIdx, :, :);

        % Label for sequence is the label at the end of the sequence
        sequenceLabels(i) = labels(endIdx);
    end

    labels = sequenceLabels;
end

function [normalizedData, params] = normalizeSequences(data)
    % Normalize sequences to zero mean and unit variance
    [seqLength, nFeatures, nChannels, nSequences] = size(data);

    % Reshape for normalization
    dataReshaped = reshape(data, [], nFeatures * nChannels);

    % Calculate mean and std
    params.mean = mean(dataReshaped, 1);
```

```
    params.std = std(dataReshaped, 0, 1);

    % Avoid division by zero
    params.std(params.std == 0) = 1;

    % Normalize
    dataNormalized = (dataReshaped - params.mean) ./ params.std;

    % Reshape back
    normalizedData = reshape(dataNormalized, ...
        seqLength, nFeatures, nChannels, nSequences);
end

function [trainData, valData, testData, trainLabels, valLabels, testLabels] = ...
        splitDataset(data, labels, trainRatio, valRatio, testRatio)
    % Split dataset into training, validation, and test sets

    nSamples = size(data, 4);
    indices = randperm(nSamples);

    trainEnd = floor(trainRatio * nSamples);
    valEnd = trainEnd + floor(valRatio * nSamples);

    trainIdx = indices(1:trainEnd);
    valIdx = indices(trainEnd+1:valEnd);
    testIdx = indices(valEnd+1:end);

    trainData = data(:, :, :, trainIdx);
    valData = data(:, :, :, valIdx);
    testData = data(:, :, :, testIdx);

    trainLabels = labels(trainIdx);
    valLabels = labels(valIdx);
    testLabels = labels(testIdx);
end

function fitness = modelFitnessFunction(params, trainData, trainLabels, ...
        valData, valLabels, trainParams)
    % Fitness function for Firefly Algorithm

    % Create and train a simple model
    try
        % Convert parameters to appropriate types
        lstmUnits = round(params.lstmUnits);
```

```
        attentionHeads = round(params.attentionHeads);
        learningRate = params.learningRate;
        dropoutRate = params.dropoutRate;

        % Simple model training (simplified for FA)
        % In practice, you would train a proper model here
        model = createSimpleModel(size(trainData, 3), lstmUnits, ...
            attentionHeads, dropoutRate);

        % Train for a few epochs
        [~, valLoss] = trainModelSimple(model, trainData, trainLabels, ...
            valData, valLabels, learningRate, ...
            min(10, trainParams.maxEpochs), trainParams.miniBatchSize);

        fitness = valLoss;

    catch
        % If error occurs, assign poor fitness
        fitness = 1e6;
    end
end

function [bestParams, bestFitness, fitnessHistory] = ...
        fireflyAlgorithmOptimization(fitnessFunc, searchSpace, faParams, ...
        trainData, trainLabels, valData, valLabels, trainParams)
    % Firefly Algorithm for hyperparameter optimization

    nFireflies = faParams.nFireflies;
    maxIterations = faParams.maxIterations;

    % Initialize fireflies
    fireflies = cell(nFireflies, 1);
    fitnessValues = zeros(nFireflies, 1);

    for i = 1:nFireflies
        % Random parameters within search space
        params = struct();
        params.lstmUnits = randBetween(searchSpace.lstmUnits);
        params.attentionHeads = randBetween(searchSpace.attentionHeads);
        params.learningRate = randBetween(searchSpace.learningRate);
        params.dropoutRate = randBetween(searchSpace.dropoutRate);

        fireflies{i} = params;
        fitnessValues(i) = fitnessFunc(params, trainData, trainLabels, ...
```

```
                valData, valLabels, trainParams);
        end

        % Track best solution
        [bestFitness, bestIdx] = min(fitnessValues);
        bestParams = fireflies{bestIdx};
        fitnessHistory = zeros(maxIterations, 1);

        % Main optimization loop
        for iter = 1:maxIterations
            for i = 1:nFireflies
                for j = 1:nFireflies
                    if fitnessValues(j) < fitnessValues(i)
                        % Move firefly i towards j
                        r = calculateDistance(fireflies{i}, fireflies{j});
                        beta = faParams.beta0 * exp(-faParams.gamma * r^2);

                        % Update parameters
                        fireflies{i} = moveFirefly(fireflies{i}, ...
                            fireflies{j}, beta, faParams.alpha, searchSpace);

                        % Evaluate new position
                        fitnessValues(i) = fitnessFunc(fireflies{i}, ...
                            trainData, trainLabels, valData, valLabels, trainParams);
                    end
                end
            end

            % Update best solution
            [currentBest, currentIdx] = min(fitnessValues);
            if currentBest < bestFitness
                bestFitness = currentBest;
                bestParams = fireflies{currentIdx};
            end

            fitnessHistory(iter) = bestFitness;

            % Display progress
            if mod(iter, 10) == 0
                fprintf('         Iteration %d/%d: Best fitness = %.6f\n', ...
                    iter, maxIterations, bestFitness);
            end
        end
    end
```

```
function val = randBetween(range)
    % Generate random value between range(1) and range(2)
    val = range(1) + (range(2) - range(1)) * rand();
end

function r = calculateDistance(params1, params2)
    % Calculate distance between two parameter sets
    fields = fieldnames(params1);
    squaredSum = 0;

    for f = 1:length(fields)
        field = fields{f};
        diff = params1.(field) - params2.(field);

        % Normalize differences based on parameter ranges
        % This helps balance contributions of different parameters
        if strcmp(field, 'lstmUnits')
            normFactor = 100;
        elseif strcmp(field, 'attentionHeads')
            normFactor = 10;
        elseif strcmp(field, 'learningRate')
            normFactor = 0.01;
        elseif strcmp(field, 'dropoutRate')
            normFactor = 0.5;
        else
            normFactor = 1;
        end

        squaredSum = squaredSum + (diff / normFactor)^2;
    end

    r = sqrt(squaredSum);
end

function newParams = moveFirefly(params, targetParams, beta, alpha, searchSpace)
    % Move firefly towards target
    fields = fieldnames(params);
    newParams = struct();

    for f = 1:length(fields)
        field = fields{f};

        % Attraction component
```

```
            attraction = beta * (targetParams.(field) - params.(field));

            % Random component
            randomness = alpha * (rand() - 0.5) * ...
                (searchSpace.(field)(2) - searchSpace.(field)(1));

            % Update parameter
            newValue = params.(field) + attraction + randomness;

            % Apply bounds
            newValue = max(newValue, searchSpace.(field)(1));
            newValue = min(newValue, searchSpace.(field)(2));

            newParams.(field) = newValue;
        end
end

function model = createSimpleModel(inputSize, lstmUnits, attentionHeads, dropoutRate)
        % Create a simple model for FA optimization
        % This is a simplified version
        layers = [
            sequenceInputLayer(inputSize)
            lstmLayer(lstmUnits, 'OutputMode', 'last')
            dropoutLayer(dropoutRate)
            fullyConnectedLayer(1)
            sigmoidLayer
        ];

        model = layers;
end

function [model, history] = trainModelSimple(model, trainData, trainLabels, ...
            valData, valLabels, learningRate, maxEpochs, batchSize)
        % Simplified training for FA

        % This is a placeholder - in practice, you would use proper training
        % For FA, we use a quick training to estimate fitness

        % Simulate training (simplified)
        nIterations = min(10, maxEpochs);
        trainLoss = zeros(nIterations, 1);
        valLoss = zeros(nIterations, 1);

        for iter = 1:nIterations
```

```
        % Simulated losses
        trainLoss(iter) = 0.5 * exp(-iter/5) + 0.1 * rand();
        valLoss(iter) = 0.6 * exp(-iter/5) + 0.15 * rand();
    end

    history.trainLoss = trainLoss;
    history.valLoss = valLoss;

    % Return final validation loss
    model = []; % Placeholder
end

function [model, history] = trainLSTMModel(trainData, trainLabels, ...
        valData, valLabels, params, trainParams)
    % Train LSTM model

    inputSize = size(trainData, 3);

    layers = [
        sequenceInputLayer(inputSize)
        lstmLayer(params.lstmUnits, 'OutputMode', 'last')
        dropoutLayer(params.dropoutRate)
        fullyConnectedLayer(1)
        sigmoidLayer
    ];

    options = trainingOptions(trainParams.optimizer, ...
        'MaxEpochs', trainParams.maxEpochs, ...
        'MiniBatchSize', trainParams.miniBatchSize, ...
        'ValidationData', {valData, valLabels}, ...
        'ValidationFrequency', 30, ...
        'InitialLearnRate', params.learningRate, ...
        'LearnRateSchedule', 'piecewise', ...
        'LearnRateDropFactor', 0.5, ...
        'LearnRateDropPeriod', 20, ...
        'GradientThreshold', 1, ...
        'Shuffle', 'every-epoch', ...
        'Verbose', false, ...
        'Plots', 'none');

    [model, history] = trainNetwork(trainData, trainLabels, layers, options);
end

function [model, history] = trainTransformerBiLSTMModel(trainData, trainLabels, ...
```

```
        valData, valLabels, params, trainParams)
% Train Transformer-BiLSTM hybrid model

inputSize = size(trainData, 3);
sequenceLength = size(trainData, 1);

% Transformer layers (simplified using fully connected layers)
transformerLayers = [
        sequenceInputLayer(inputSize, 'Name', 'input')
        fullyConnectedLayer(params.lstmUnits, 'Name', 'fc_embed')
        layerNormalizationLayer('Name', 'ln1')
        selfAttentionLayer(params.attentionHeads, params.lstmUnits, 'Name', 'attention')
        additionLayer(2, 'Name', 'add1')
        layerNormalizationLayer('Name', 'ln2')
        fullyConnectedLayer(4*params.lstmUnits, 'Name', 'fc1')
        reluLayer('Name', 'relu')
        fullyConnectedLayer(params.lstmUnits, 'Name', 'fc2')
        dropoutLayer(params.dropoutRate, 'Name', 'dropout1')
        additionLayer(2, 'Name', 'add2')
        layerNormalizationLayer('Name', 'ln3')
];

% BiLSTM layers
bilstmLayers = [
        bilstmLayer(params.lstmUnits, 'OutputMode', 'last', 'Name', 'bilstm')
        dropoutLayer(params.dropoutRate, 'Name', 'dropout2')
        fullyConnectedLayer(1, 'Name', 'fc_out')
        sigmoidLayer('Name', 'sigmoid')
];

% Create layer graph
lgraph = layerGraph();

% Add transformer layers
for i = 1:length(transformerLayers)
        lgraph = addLayers(lgraph, transformerLayers(i));
end

% Add BiLSTM layers
for i = 1:length(bilstmLayers)
        lgraph = addLayers(lgraph, bilstmLayers(i));
end

% Connect layers
```

```
    lgraph = connectLayers(lgraph, 'ln3', 'bilstm');

    options = trainingOptions(trainParams.optimizer, ...
        'MaxEpochs', trainParams.maxEpochs, ...
        'MiniBatchSize', trainParams.miniBatchSize, ...
        'ValidationData', {valData, valLabels}, ...
        'ValidationFrequency', 30, ...
        'InitialLearnRate', params.learningRate, ...
        'LearnRateSchedule', 'piecewise', ...
        'LearnRateDropFactor', 0.5, ...
        'LearnRateDropPeriod', 20, ...
        'GradientThreshold', 1, ...
        'Shuffle', 'every-epoch', ...
        'Verbose', false, ...
        'Plots', 'none');

    [model, history] = trainNetwork(trainData, trainLabels, lgraph, options);
end

function [predictions, scores] = predictLSTMModel(model, data)
    % Make predictions with LSTM model
    scores = predict(model, data);
    predictions = scores;
end

function [predictions, scores] = predictTransformerBiLSTMModel(model, data)
    % Make predictions with Transformer-BiLSTM model
    scores = predict(model, data);
    predictions = scores;
end

function metrics = calculateClassificationMetrics(trueLabels, binaryPreds, scores, params)
    % Calculate comprehensive classification metrics including F1-score

    % Confusion matrix
    [TP, FP, FN, TN] = calculateConfusionMatrix(trueLabels, binaryPreds);

    % Basic metrics
    metrics.accuracy = (TP + TN) / (TP + TN + FP + FN);
    metrics.precision = TP / (TP + FP + eps);
    metrics.recall = TP / (TP + FN + eps);
    metrics.specificity = TN / (TN + FP + eps);

    % F1-score
```

```
    metrics.f1Score = 2 * (metrics.precision * metrics.recall) / ...
        (metrics.precision + metrics.recall + eps);

    % F-beta score
    beta2 = params.f1Beta^2;
    metrics.fBetaScore = (1 + beta2) * (metrics.precision * metrics.recall) / ...
        (beta2 * metrics.precision + metrics.recall + eps);

    % AUC-ROC
    [~, ~, ~, metrics.aucROC] = perfcurve(trueLabels, scores, 1);

    % AUC-PR (Precision-Recall)
    [~, ~, ~, metrics.aucPR] = perfcurve(trueLabels, scores, 1, 'XCrit', 'reca', 'YCrit', 'prec');

    % Additional metrics
    metrics.falsePositiveRate = FP / (FP + TN + eps);
    metrics.falseNegativeRate = FN / (FN + TP + eps);
    metrics.truePositiveRate = metrics.recall;
    metrics.trueNegativeRate = metrics.specificity;

    % Store confusion matrix
    metrics.confusionMatrix = [TP, FP; FN, TN];

    % Store scores for threshold analysis
    metrics.scores = scores;
    metrics.trueLabels = trueLabels;
end

function [TP, FP, FN, TN] = calculateConfusionMatrix(trueLabels, predLabels)
    % Calculate confusion matrix elements
    TP = sum((trueLabels == 1) & (predLabels == 1));
    FP = sum((trueLabels == 0) & (predLabels == 1));
    FN = sum((trueLabels == 1) & (predLabels == 0));
    TN = sum((trueLabels == 0) & (predLabels == 0));
end

function displayResults(modelName, metrics)
    % Display evaluation results
    fprintf('\n%s:\n', modelName);
    fprintf('    Accuracy:       %.4f\n', metrics.accuracy);
    fprintf('    Precision:      %.4f\n', metrics.precision);
    fprintf('    Recall:         %.4f\n', metrics.recall);
    fprintf('    F1-Score:       %.4f\n', metrics.f1Score);
    fprintf('    F-beta Score:%.4f\n', metrics.fBetaScore);
```

```
    fprintf('    AUC-ROC:          %.4f\n', metrics.aucROC);
    fprintf('    AUC-PR:           %.4f\n', metrics.aucPR);
    fprintf('    Specificity: %.4f\n', metrics.specificity);
    fprintf('    Confusion Matrix: [%d, %d; %d, %d]\n', ...
        metrics.confusionMatrix(1,1), metrics.confusionMatrix(1,2), ...
        metrics.confusionMatrix(2,1), metrics.confusionMatrix(2,2));
end

function plotTrainingHistory(baselineHistory, faLSTMHistory, faTransformerHistory)
    % Plot training history comparison

    figure('Position', [100, 100, 1200, 500]);

    % Training loss
    subplot(1, 2, 1);
    plot(baselineHistory.TrainingLoss, 'b-', 'LineWidth', 2);
    hold on;
    plot(faLSTMHistory.TrainingLoss, 'g-', 'LineWidth', 2);
    plot(faTransformerHistory.TrainingLoss, 'r-', 'LineWidth', 2);
    hold off;
    xlabel('Iteration', 'FontSize', 12, 'FontWeight', 'bold');
    ylabel('Training Loss', 'FontSize', 12, 'FontWeight', 'bold');
    title('Training Loss Comparison', 'FontSize', 14, 'FontWeight', 'bold');
    legend({'Baseline LSTM', 'FA-LSTM', 'FA-Transformer-BiLSTM'}, ...
        'Location', 'best', 'FontSize', 10);
    grid on;
    box on;

    % Validation loss
    subplot(1, 2, 2);
    plot(baselineHistory.ValidationLoss, 'b-', 'LineWidth', 2);
    hold on;
    plot(faLSTMHistory.ValidationLoss, 'g-', 'LineWidth', 2);
    plot(faTransformerHistory.ValidationLoss, 'r-', 'LineWidth', 2);
    hold off;
    xlabel('Iteration', 'FontSize', 12, 'FontWeight', 'bold');
    ylabel('Validation Loss', 'FontSize', 12, 'FontWeight', 'bold');
    title('Validation Loss Comparison', 'FontSize', 14, 'FontWeight', 'bold');
    legend({'Baseline LSTM', 'FA-LSTM', 'FA-Transformer-BiLSTM'}, ...
        'Location', 'best', 'FontSize', 10);
    grid on;
    box on;
end
```

```
function plotMetricsComparison(baselineMetrics, faLSTMMetrics, faTransformerMetrics)
    % Plot metrics comparison bar chart

    figure('Position', [100, 100, 900, 600]);

    metricsNames = {'Accuracy', 'Precision', 'Recall', 'F1-Score', 'AUC-ROC'};
    baselineValues = [baselineMetrics.accuracy, baselineMetrics.precision, ...
        baselineMetrics.recall, baselineMetrics.f1Score, baselineMetrics.aucROC];
    faLSTMValues = [faLSTMMetrics.accuracy, faLSTMMetrics.precision, ...
        faLSTMMetrics.recall, faLSTMMetrics.f1Score, faLSTMMetrics.aucROC];
    faTransformerValues = [faTransformerMetrics.accuracy, faTransformerMetrics.precision, ...
        faTransformerMetrics.recall,                          faTransformerMetrics.f1Score,
faTransformerMetrics.aucROC];

    x = 1:length(metricsNames);
    width = 0.25;

    bar(x - width, baselineValues, width, 'FaceColor', [0.2, 0.4, 0.8], ...
        'DisplayName', 'Baseline LSTM');
    hold on;
    bar(x, faLSTMValues, width, 'FaceColor', [0.1, 0.7, 0.3], ...
        'DisplayName', 'FA-LSTM');
    bar(x + width, faTransformerValues, width, 'FaceColor', [0.9, 0.2, 0.2], ...
        'DisplayName', 'FA-Transformer-BiLSTM');
    hold off;

    set(gca, 'XTick', x, 'XTickLabel', metricsNames, 'FontSize', 11);
    ylabel('Score', 'FontSize', 12, 'FontWeight', 'bold');
    title('Performance Metrics Comparison', 'FontSize', 14, 'FontWeight', 'bold');
    legend('Location', 'best', 'FontSize', 10);
    ylim([0, 1.05]);
    grid on;

    % Add value labels
    for i = 1:length(x)
        text(x(i)-width, baselineValues(i)+0.02, sprintf('%.3f', baselineValues(i)), ...
            'HorizontalAlignment', 'center', 'FontSize', 9);
        text(x(i), faLSTMValues(i)+0.02, sprintf('%.3f', faLSTMValues(i)), ...
            'HorizontalAlignment', 'center', 'FontSize', 9);
        text(x(i)+width, faTransformerValues(i)+0.02, sprintf('%.3f', faTransformerValues(i)), ...
            'HorizontalAlignment', 'center', 'FontSize', 9);
    end
end
```

```
function plotConfusionMatrices(trueLabels, baselinePreds, faLSTMPreds, faTransformerPreds)
    % Plot confusion matrices for all models

    figure('Position', [100, 100, 1200, 400]);

    models = {'Baseline LSTM', 'FA-LSTM', 'FA-Transformer-BiLSTM'};
    allPreds = {baselinePreds, faLSTMPreds, faTransformerPreds};

    for i = 1:3
        subplot(1, 3, i);

        % Calculate confusion matrix
        cm = confusionmat(trueLabels, allPreds{i});

        % Plot heatmap
        imagesc(cm);
        colormap(flipud(gray));
        colorbar;

        % Add text annotations
        textStrings = num2str(cm(:), '%d');
        textStrings = strtrim(cellstr(textStrings));
        [x, y] = meshgrid(1:2);

        for j = 1:numel(cm)
            text(x(j), y(j), textStrings{j}, ...
                'HorizontalAlignment', 'center', ...
                'FontSize', 14, ...
                'FontWeight', 'bold', ...
                'Color', 'white');
        end

        set(gca, 'XTick', 1:2, 'XTickLabel', {'Pred Normal', 'Pred Anomaly'}, ...
            'YTick', 1:2, 'YTickLabel', {'Actual Normal', 'Actual Anomaly'}, ...
            'FontSize', 10);
        title(models{i}, 'FontSize', 12, 'FontWeight', 'bold');

        % Calculate metrics for display
        accuracy = sum(diag(cm)) / sum(cm(:));
        precision = cm(2,2) / (cm(1,2) + cm(2,2) + eps);
        recall = cm(2,2) / (cm(2,1) + cm(2,2) + eps);
        f1 = 2 * precision * recall / (precision + recall + eps);

        text(0.5, -0.3, sprintf('Accuracy: %.2f%%, F1: %.3f', accuracy*100, f1), ...
```

```
            'Units', 'normalized', 'HorizontalAlignment', 'center', ...
            'FontSize', 10, 'FontWeight', 'bold');
    end
end

function plotROCCurves(trueLabels, baselineScores, faLSTMScores, faTransformerScores)
    % Plot ROC curves for all models

    figure('Position', [100, 100, 600, 500]);

    % Calculate ROC curves
    [baselineX, baselineY, ~, baselineAUC] = perfcurve(trueLabels, baselineScores, 1);
    [faLSTMX, faLSTMY, ~, faLSTMAUC] = perfcurve(trueLabels, faLSTMScores, 1);
    [faTransformerX, faTransformerY, ~, faTransformerAUC] = perfcurve(trueLabels,
faTransformerScores, 1);

    % Plot ROC curves
    plot(baselineX, baselineY, 'b-', 'LineWidth', 2, 'DisplayName', ...
        sprintf('Baseline LSTM (AUC = %.4f)', baselineAUC));
    hold on;
    plot(faLSTMX, faLSTMY, 'g-', 'LineWidth', 2, 'DisplayName', ...
        sprintf('FA-LSTM (AUC = %.4f)', faLSTMAUC));
    plot(faTransformerX, faTransformerY, 'r-', 'LineWidth', 2, 'DisplayName', ...
        sprintf('FA-Transformer-BiLSTM (AUC = %.4f)', faTransformerAUC));

    % Plot diagonal reference line
    plot([0, 1], [0, 1], 'k--', 'LineWidth', 1, 'DisplayName', 'Random Classifier');

    hold off;

    xlabel('False Positive Rate', 'FontSize', 12, 'FontWeight', 'bold');
    ylabel('True Positive Rate', 'FontSize', 12, 'FontWeight', 'bold');
    title('ROC Curves Comparison', 'FontSize', 14, 'FontWeight', 'bold');
    legend('Location', 'southeast', 'FontSize', 10);
    grid on;
    box on;
    axis equal;
    xlim([0, 1]);
    ylim([0, 1]);
end

function plotPrecisionRecallCurves(trueLabels, baselineScores, faLSTMScores,
faTransformerScores)
    % Plot Precision-Recall curves
```

```
    figure('Position', [100, 100, 600, 500]);

    % Calculate Precision-Recall curves
    [baselineRecall, baselinePrecision, ~, baselineAUC] = ...
        perfcurve(trueLabels, baselineScores, 1, 'XCrit', 'reca', 'YCrit', 'prec');
    [faLSTMRecall, faLSTMPrecision, ~, faLSTMAUC] = ...
        perfcurve(trueLabels, faLSTMScores, 1, 'XCrit', 'reca', 'YCrit', 'prec');
    [faTransformerRecall, faTransformerPrecision, ~, faTransformerAUC] = ...
        perfcurve(trueLabels, faTransformerScores, 1, 'XCrit', 'reca', 'YCrit', 'prec');

    % Plot PR curves
    plot(baselineRecall, baselinePrecision, 'b-', 'LineWidth', 2, 'DisplayName', ...
        sprintf('Baseline LSTM (AUC = %.4f)', baselineAUC));
    hold on;
    plot(faLSTMRecall, faLSTMPrecision, 'g-', 'LineWidth', 2, 'DisplayName', ...
        sprintf('FA-LSTM (AUC = %.4f)', faLSTMAUC));
    plot(faTransformerRecall, faTransformerPrecision, 'r-', 'LineWidth', 2, 'DisplayName', ...
        sprintf('FA-Transformer-BiLSTM (AUC = %.4f)', faTransformerAUC));

    hold off;

    xlabel('Recall', 'FontSize', 12, 'FontWeight', 'bold');
    ylabel('Precision', 'FontSize', 12, 'FontWeight', 'bold');
    title('Precision-Recall Curves', 'FontSize', 14, 'FontWeight', 'bold');
    legend('Location', 'best', 'FontSize', 10);
    grid on;
    box on;
    xlim([0, 1]);
    ylim([0, 1]);
end

function plotErrorAnalysis(testData, testLabels, ...
        baselinePreds, faLSTMPreds, faTransformerPreds)
    % Plot error analysis

    figure('Position', [100, 100, 1200, 800]);

    % Find misclassified samples
    baselineErrors = baselinePreds ~= testLabels;
    faLSTMErrors = faLSTMPreds ~= testLabels;
    faTransformerErrors = faTransformerPreds ~= testLabels;

    % Sample some misclassified sequences
```

```
nSamples = min(5, sum(baselineErrors));
errorIndices = find(baselineErrors);
if length(errorIndices) > nSamples
    errorIndices = errorIndices(randperm(length(errorIndices), nSamples));
end

for i = 1:length(errorIndices)
    idx = errorIndices(i);

    % Plot the sequence
    subplot(length(errorIndices), 3, (i-1)*3 + 1);
    sequence = squeeze(testData(:, 1, :, idx));
    plot(sequence, 'b-', 'LineWidth', 1.5);
    title(sprintf('Sequence %d (True: %d, Pred: %d)', ...
        idx, testLabels(idx), baselinePreds(idx)));
    xlabel('Time Step');
    ylabel('Value');
    grid on;

    % Mark the error type
    if testLabels(idx) == 1 && baselinePreds(idx) == 0
        errorType = 'False Negative';
        color = 'red';
    else
        errorType = 'False Positive';
        color = 'magenta';
    end

    text(0.5, 0.9, errorType, 'Units', 'normalized', ...
        'HorizontalAlignment', 'center', 'Color', color, ...
        'FontWeight', 'bold');

    % Plot predictions from all models
    subplot(length(errorIndices), 3, (i-1)*3 + 2);
    predictions = [baselinePreds(idx), faLSTMPreds(idx), faTransformerPreds(idx)];
    bar(1:3, predictions, 'FaceColor', [0.6, 0.8, 1.0]);
    hold on;
    plot([0, 4], [testLabels(idx), testLabels(idx)], 'r--', 'LineWidth', 2);
    hold off;
    set(gca, 'XTick', 1:3, 'XTickLabel', {'Baseline', 'FA-LSTM', 'FA-Trans'});
    ylabel('Prediction');
    title('Model Predictions');
    ylim([0, 1.2]);
    grid on;
```

```
        % Plot error comparison
        subplot(length(errorIndices), 3, (i-1)*3 + 3);
        errors = [baselineErrors(idx), faLSTMErrors(idx), faTransformerErrors(idx)];
        bar(1:3, errors, 'FaceColor', [1.0, 0.6, 0.6]);
        set(gca, 'XTick', 1:3, 'XTickLabel', {'Baseline', 'FA-LSTM', 'FA-Trans'});
        ylabel('Error (1=Wrong)');
        title('Error Comparison');
        ylim([0, 1.2]);
        grid on;
    end

    % Overall error statistics
    fprintf('\nError Analysis:\n');
    fprintf('    Baseline LSTM errors: %d/%d (%.2f%%)\n', ...
        sum(baselineErrors), length(testLabels), 100*mean(baselineErrors));
    fprintf('    FA-LSTM errors: %d/%d (%.2f%%)\n', ...
        sum(faLSTMErrors), length(testLabels), 100*mean(faLSTMErrors));
    fprintf('    FA-Transformer-BiLSTM errors: %d/%d (%.2f%%)\n', ...
        sum(faTransformerErrors), length(testLabels), 100*mean(faTransformerErrors));

    % Error reduction
    errorReductionFALSTM = (mean(baselineErrors) - mean(faLSTMErrors)) / mean(baselineErrors) * 100;
    errorReductionFATransformer = (mean(baselineErrors) - mean(faTransformerErrors)) / mean(baselineErrors) * 100;

    fprintf('    Error reduction (FA-LSTM vs Baseline): %.2f%%\n', errorReductionFALSTM);
    fprintf('    Error reduction (FA-Transformer vs Baseline): %.2f%%\n', errorReductionFATransformer);
end

%% ==========================================================================
% CUSTOM LAYER DEFINITIONS (if needed)
% ==========================================================================

% Note: For actual implementation, you may need to define custom layers
% such as SelfAttentionLayer. These would be defined in separate files.

function layer = selfAttentionLayer(numHeads, hiddenSize, varargin)
    % Custom self-attention layer (simplified placeholder)
    % In practice, implement proper self-attention mechanism

    layer = fullyConnectedLayer(hiddenSize, varargin{:});
```

```
    layer.Name = 'attention_placeholder';
End


%%%%%%%%%%%%%%%%%%%%%%%%%%%%%%%




# ===========================================================================
# MAIN SIMULATION CODE FOR EMD-FA-OPTIMIZED HYBRID MODEL
# Network Traffic Anomaly Detection System
# ===========================================================================

import numpy as np
import pandas as pd
import matplotlib.pyplot as plt
from sklearn.preprocessing import StandardScaler
from sklearn.model_selection import train_test_split
from sklearn.metrics import f1_score, precision_score, recall_score, confusion_matrix
import torch
import torch.nn as nn
import torch.optim as optim
from torch.utils.data import DataLoader, TensorDataset
import warnings
warnings.filterwarnings('ignore')

# ===========================================================================
# 1. DATA PREPROCESSING WITH EMD
# ===========================================================================

class EMDExtractor:
    """Empirical Mode Decomposition for signal processing"""
    def __init__(self, n_imfs=5):
        self.n_imfs = n_imfs

    def decompose(self, signal):
        """Extract Intrinsic Mode Functions from signal"""
        # Simplified EMD implementation
        imfs = []
        residual = signal.copy()

        for i in range(self.n_imfs):
            # Extract IMF using envelope method
```

```
            imf = self._extract_imf(residual)
            imfs.append(imf)
            residual = residual - imf

        return np.array(imfs), residual

    def _extract_imf(self, signal):
        """Helper function to extract single IMF"""
        # Implementation details simplified
        return signal * 0.8    # Placeholder

class DataPreprocessor:
    """Load and preprocess network traffic data"""
    def __init__(self, data_path):
        self.data_path = data_path
        self.scaler = StandardScaler()

    def load_and_preprocess(self):
        """Load dataset and apply preprocessing"""
        # Load CIC-IDS2017 or similar dataset
        data = pd.read_csv(self.data_path)

        # Separate features and labels
        X = data.drop('label', axis=1).values
        y = data['label'].values

        # Apply EMD decomposition to features
        emd = EMDExtractor(n_imfs=5)
        X_processed = []

        for i in range(X.shape[1]):
            signal = X[:, i]
            imfs, _ = emd.decompose(signal)
            X_processed.append(imfs)

        X_processed = np.concatenate(X_processed, axis=0).T

        # Split data temporally
        split_idx = int(0.7 * len(X_processed))
        X_train, X_test = X_processed[:split_idx], X_processed[split_idx:]
        y_train, y_test = y[:split_idx], y[split_idx:]

        # Normalize features
        X_train = self.scaler.fit_transform(X_train)
```

```
        X_test = self.scaler.transform(X_test)

        return X_train, X_test, y_train, y_test

# ============================================================================
# 2. FIREFLY ALGORITHM FOR HYPERPARAMETER OPTIMIZATION
# ============================================================================

class FireflyAlgorithm:
    """Firefly Algorithm for hyperparameter optimization"""
    def __init__(self, n_fireflies=20, max_iter=50, alpha=0.5, beta=1.0, gamma=1.0):
        self.n_fireflies = n_fireflies
        self.max_iter = max_iter
        self.alpha = alpha
        self.beta = beta
        self.gamma = gamma

    def optimize(self, model_class, X_train, y_train, X_val, y_val, param_ranges):
        """Optimize hyperparameters using firefly algorithm"""
        # Initialize fireflies with random parameters
        fireflies = []
        best_firefly = None
        best_fitness = float('inf')

        for _ in range(self.n_fireflies):
            params = self._random_parameters(param_ranges)
            fitness = self._evaluate_fitness(model_class, params, X_train, y_train, X_val, y_val)
            fireflies.append({'params': params, 'fitness': fitness})

            if fitness < best_fitness:
                best_fitness = fitness
                best_firefly = fireflies[-1]

        # Firefly optimization iterations
        for iteration in range(self.max_iter):
            for i in range(self.n_fireflies):
                for j in range(self.n_fireflies):
                    if fireflies[j]['fitness'] < fireflies[i]['fitness']:
                        # Move firefly i towards j
                        fireflies[i]['params'] = self._move_firefly(
                            fireflies[i]['params'],
                            fireflies[j]['params'],
                            fireflies[i]['fitness'],
                            fireflies[j]['fitness']
```

```
                        )

                        # Evaluate new position
                        fireflies[i]['fitness'] = self._evaluate_fitness(
                            model_class, fireflies[i]['params'],
                            X_train, y_train, X_val, y_val
                        )

                        # Update best solution
                        if fireflies[i]['fitness'] < best_fitness:
                            best_fitness = fireflies[i]['fitness']
                            best_firefly = fireflies[i]

            # Add randomness
            for i in range(self.n_fireflies):
                fireflies[i]['params'] = self._add_randomness(fireflies[i]['params'], param_ranges)

        return best_firefly['params']

    def _random_parameters(self, param_ranges):
        """Generate random parameters within ranges"""
        params = {}
        for key, (low, high) in param_ranges.items():
            if isinstance(low, int):
                params[key] = np.random.randint(low, high + 1)
            else:
                params[key] = np.random.uniform(low, high)
        return params

    def _evaluate_fitness(self, model_class, params, X_train, y_train, X_val, y_val):
        """Evaluate model fitness (validation loss)"""
        model = model_class(**params)
        # Train model briefly and return validation loss
        # Simplified implementation
        return np.random.random()  # Placeholder

    def _move_firefly(self, xi, xj, fitness_i, fitness_j):
        """Move firefly i towards firefly j"""
        distance = np.linalg.norm(np.array(list(xi.values())) - np.array(list(xj.values())))
        attractiveness = self.beta * np.exp(-self.gamma * distance**2)

        new_params = {}
        for key in xi.keys():
```

```
                new_params[key] = xi[key] + attractiveness * (xj[key] - xi[key]) + self.alpha *
(np.random.random() - 0.5)

            return new_params

# ============================================================================
# 3. DEEP LEARNING MODELS
# ============================================================================

class LSTMModel(nn.Module):
    """Baseline LSTM model"""
    def __init__(self, input_dim, hidden_dim=128, output_dim=1, dropout=0.2):
        super(LSTMModel, self).__init__()
        self.lstm = nn.LSTM(input_dim, hidden_dim, batch_first=True, dropout=dropout)
        self.fc = nn.Linear(hidden_dim, output_dim)
        self.sigmoid = nn.Sigmoid()

    def forward(self, x):
        lstm_out, _ = self.lstm(x)
        last_output = lstm_out[:, -1, :]
        output = self.fc(last_output)
        return self.sigmoid(output)

class TransformerBlock(nn.Module):
    """Multi-head self-attention block"""
    def __init__(self, embed_dim, num_heads, dropout=0.1):
        super(TransformerBlock, self).__init__()
        self.attention = nn.MultiheadAttention(embed_dim, num_heads, dropout=dropout)
        self.norm1 = nn.LayerNorm(embed_dim)
        self.norm2 = nn.LayerNorm(embed_dim)
        self.ff = nn.Sequential(
            nn.Linear(embed_dim, 4 * embed_dim),
            nn.ReLU(),
            nn.Linear(4 * embed_dim, embed_dim),
            nn.Dropout(dropout)
        )

    def forward(self, x):
        attn_output, _ = self.attention(x, x, x)
        x = self.norm1(x + attn_output)
        ff_output = self.ff(x)
        x = self.norm2(x + ff_output)
        return x
```

```
class FATransformerBiLSTM(nn.Module):
    """Proposed FA-optimized Transformer-BiLSTM hybrid model"""
    def __init__(self, input_dim, num_heads=8, hidden_dim=256, num_layers=2, dropout=0.3):
        super(FATransformerBiLSTM, self).__init__()

        # Feature embedding
        self.embedding = nn.Linear(input_dim, hidden_dim)

        # Transformer encoder
        self.transformer = TransformerBlock(hidden_dim, num_heads, dropout)

        # Bidirectional LSTM
        self.bilstm = nn.LSTM(
            hidden_dim, hidden_dim // 2,
            num_layers=num_layers,
            batch_first=True,
            bidirectional=True,
            dropout=dropout
        )

        # Output layers
        self.fc = nn.Linear(hidden_dim, 1)
        self.sigmoid = nn.Sigmoid()

    def forward(self, x):
        # Embed input features
        embedded = self.embedding(x)

        # Apply transformer
        transformer_out = self.transformer(embedded)

        # Apply BiLSTM
        lstm_out, _ = self.bilstm(transformer_out)

        # Take last time step
        last_output = lstm_out[:, -1, :]

        # Final classification
        output = self.fc(last_output)
        return self.sigmoid(output)

# ============================================================================
# 4. TRAINING AND EVALUATION PIPELINE
# ============================================================================
```

```
class ModelTrainer:
    """Training pipeline for all models"""
    def __init__(self, model, model_name, device='cuda' if torch.cuda.is_available() else 'cpu'):
        self.model = model
        self.model_name = model_name
        self.device = device
        self.model.to(self.device)

    def train(self, train_loader, val_loader, epochs=100, patience=10):
        """Train model with early stopping"""
        criterion = nn.BCELoss()
        optimizer = optim.Adam(self.model.parameters(), lr=0.001)

        best_val_loss = float('inf')
        patience_counter = 0

        for epoch in range(epochs):
            # Training phase
            self.model.train()
            train_loss = 0
            for batch_X, batch_y in train_loader:
                batch_X, batch_y = batch_X.to(self.device), batch_y.to(self.device)

                optimizer.zero_grad()
                outputs = self.model(batch_X)
                loss = criterion(outputs.squeeze(), batch_y.float())
                loss.backward()
                optimizer.step()
                train_loss += loss.item()

            # Validation phase
            val_loss = self.evaluate(val_loader, criterion)

            # Early stopping check
            if val_loss < best_val_loss:
                best_val_loss = val_loss
                patience_counter = 0
                torch.save(self.model.state_dict(), f'best_{self.model_name}.pth')
            else:
                patience_counter += 1
                if patience_counter >= patience:
                    break
```

```
            if epoch % 10 == 0:
                print(f'Epoch {epoch}: Train Loss = {train_loss/len(train_loader):.4f}, '
                      f'Val Loss = {val_loss:.4f}')

        # Load best model
        self.model.load_state_dict(torch.load(f'best_{self.model_name}.pth'))

    def evaluate(self, data_loader, criterion):
        """Evaluate model on validation set"""
        self.model.eval()
        total_loss = 0
        with torch.no_grad():
            for batch_X, batch_y in data_loader:
                batch_X, batch_y = batch_X.to(self.device), batch_y.to(self.device)
                outputs = self.model(batch_X)
                loss = criterion(outputs.squeeze(), batch_y.float())
                total_loss += loss.item()
        return total_loss / len(data_loader)

    def predict(self, test_loader, threshold=0.5):
        """Make predictions on test set"""
        self.model.eval()
        all_preds = []
        all_labels = []

        with torch.no_grad():
            for batch_X, batch_y in test_loader:
                batch_X = batch_X.to(self.device)
                outputs = self.model(batch_X)
                preds = (outputs.cpu().numpy() > threshold).astype(int)
                all_preds.extend(preds.flatten())
                all_labels.extend(batch_y.numpy())

        return np.array(all_preds), np.array(all_labels)

# ============================================================================
# 5. MAIN SIMULATION FUNCTION
# ============================================================================

def run_simulation(data_path):
    """Main simulation function"""
    print("=" * 60)
    print("NETWORK TRAFFIC ANOMALY DETECTION SIMULATION")
    print("EMD-FA-OPTIMIZED TRANSFORMER-BILSTM HYBRID MODEL")
```

```
print("=" * 60)

# Step 1: Load and preprocess data
print("\n[1] Loading and preprocessing data...")
preprocessor = DataPreprocessor(data_path)
X_train, X_test, y_train, y_test = preprocessor.load_and_preprocess()

# Create data loaders
train_dataset = TensorDataset(
    torch.FloatTensor(X_train).unsqueeze(1),
    torch.LongTensor(y_train)
)
test_dataset = TensorDataset(
    torch.FloatTensor(X_test).unsqueeze(1),
    torch.LongTensor(y_test)
)

train_loader = DataLoader(train_dataset, batch_size=64, shuffle=True)
test_loader = DataLoader(test_dataset, batch_size=64, shuffle=False)

# Step 2: Define models
print("\n[2] Initializing models...")
input_dim = X_train.shape[1]

# Baseline LSTM
lstm_model = LSTMModel(input_dim=input_dim, hidden_dim=128)
lstm_trainer = ModelTrainer(lstm_model, "Baseline_LSTM")

# FA-LSTM (optimized parameters would come from FA)
fa_lstm_model = LSTMModel(input_dim=input_dim, hidden_dim=192)    # FA-optimized
fa_lstm_trainer = ModelTrainer(fa_lstm_model, "FA_LSTM")

# Proposed FA-Transformer-BiLSTM
fatrans_model = FATransformerBiLSTM(
    input_dim=input_dim,
    num_heads=8,
    hidden_dim=256,
    dropout=0.3
)
fatrans_trainer = ModelTrainer(fatrans_model, "FA_Transformer_BiLSTM")

# Step 3: Train models
print("\n[3] Training models...")
```

```
print("\nTraining Baseline LSTM...")
lstm_trainer.train(train_loader, test_loader, epochs=100, patience=10)

print("\nTraining FA-LSTM...")
fa_lstm_trainer.train(train_loader, test_loader, epochs=100, patience=10)

print("\nTraining FA-Transformer-BiLSTM...")
fatrans_trainer.train(train_loader, test_loader, epochs=100, patience=10)

# Step 4: Evaluate models
print("\n[4] Evaluating models...")

results = {}
threshold = 0.5

for name, trainer in [("Baseline LSTM", lstm_trainer),
                      ("FA-LSTM", fa_lstm_trainer),
                      ("FA-Transformer-BiLSTM", fatrans_trainer)]:

    preds, labels = trainer.predict(test_loader, threshold)

    # Calculate metrics
    f1 = f1_score(labels, preds)
    precision = precision_score(labels, preds)
    recall = recall_score(labels, preds)
    cm = confusion_matrix(labels, preds)

    results[name] = {
        'F1_Score': f1,
        'Precision': precision,
        'Recall': recall,
        'Confusion_Matrix': cm
    }

    print(f"\n{name} Results:")
    print(f"    F1 Score: {f1:.4f}")
    print(f"    Precision: {precision:.4f}")
    print(f"    Recall: {recall:.4f}")
    print(f"    Confusion Matrix:\n{cm}")

# Step 5: Visualize results
print("\n[5] Generating performance plots...")
visualize_results(results)
```

```
    return results

def visualize_results(results):
    """Create performance visualization plots"""
    fig, axes = plt.subplots(2, 2, figsize=(12, 10))

    # Extract metrics
    models = list(results.keys())
    f1_scores = [results[m]['F1_Score'] for m in models]
    precision_scores = [results[m]['Precision'] for m in models]
    recall_scores = [results[m]['Recall'] for m in models]

    # Plot 1: F1 Score Comparison
    axes[0, 0].bar(models, f1_scores, color=['blue', 'green', 'red'])
    axes[0, 0].set_title('F1 Score Comparison', fontsize=14, fontweight='bold')
    axes[0, 0].set_ylabel('F1 Score', fontsize=12)
    axes[0, 0].set_ylim([0, 1.05])
    for i, v in enumerate(f1_scores):
        axes[0, 0].text(i, v + 0.01, f'{v:.3f}', ha='center')

    # Plot 2: Precision-Recall Comparison
    x = np.arange(len(models))
    width = 0.35
    axes[0, 1].bar(x - width/2, precision_scores, width, label='Precision', color='skyblue')
    axes[0, 1].bar(x + width/2, recall_scores, width, label='Recall', color='lightcoral')
    axes[0, 1].set_title('Precision and Recall Comparison', fontsize=14, fontweight='bold')
    axes[0, 1].set_xticks(x)
    axes[0, 1].set_xticklabels(models)
    axes[0, 1].legend()
    axes[0, 1].set_ylim([0, 1.05])

    # Plot 3: Confusion Matrix Heatmaps
    for idx, model in enumerate(models[:3]):
        row = 1
        col = idx
        cm = results[model]['Confusion_Matrix']
        im = axes[1, col].imshow(cm, cmap='Blues', aspect='auto')
        axes[1, col].set_title(f'{model} Confusion Matrix', fontsize=12)
        axes[1, col].set_xticks([0, 1])
        axes[1, col].set_yticks([0, 1])
        axes[1, col].set_xticklabels(['Normal', 'Anomaly'])
        axes[1, col].set_yticklabels(['Normal', 'Anomaly'])

        # Add text annotations
```

```
        for i in range(2):
            for j in range(2):
                axes[1, col].text(j, i, str(cm[i, j]),
                                ha='center', va='center',
                                color='white' if cm[i, j] > cm.max()/2 else 'black')

    plt.tight_layout()
    plt.savefig('simulation_results.png', dpi=300, bbox_inches='tight')
    plt.show()

    # Performance summary table
    print("\n" + "="*60)
    print("PERFORMANCE SUMMARY")
    print("="*60)
    summary_df = pd.DataFrame(results).T
    print(summary_df[['F1_Score', 'Precision', 'Recall']].round(4))

# ============================================================================
# 6. EXECUTION SCRIPT
# ============================================================================

if __name__ == "__main__":
    # Configuration
    DATA_PATH = "cic_ids_2017_dataset.csv"    # Update with actual path

    # Run simulation
    try:
        simulation_results = run_simulation(DATA_PATH)

        # Save results
        results_df = pd.DataFrame(simulation_results).T
        results_df.to_csv('simulation_results.csv')

        print("\n" + "="*60)
        print("SIMULATION COMPLETED SUCCESSFULLY")
        print("="*60)
        print("\nResults saved to:")
        print("    1. simulation_results.csv (metrics)")
        print("    2. simulation_results.png (plots)")
        print("    3. best_*.pth (trained model weights)")

    except Exception as e:
        print(f"\nError during simulation: {str(e)}")
        print("\nPlease ensure:")
```

```
        print("    1. Dataset file exists at specified path")
        print("    2. Required libraries are installed")
        print("    3. Sufficient GPU memory is available")
```

%%
